# Magneto‐Sono‐Enhanced Nanozyme Catalysis Drives ROS‐Mediated Pathogen Killing and Mitophagy‐Linked Inflammation Control in Bacterial Pneumonia

**DOI:** 10.1002/advs.77785

**Published:** 2026-09-16

**Authors:** Xiaofeng Luo, Yundi Wu, Shuai Zhang, Huanran Qu, Shiyang Shao, Jianqiang Chen, Xilong Wu

**Affiliations:** ^1^ State Key Laboratory of Digital Medical Engineering School of Biomedical Engineering The Affiliated Sanya Central Hospital of Hainan University School of Pharmaceutical Sciences Hainan University Sanya China; ^2^ Collaborative Innovation Center of One Health, Key Laboratory of Biomedical Engineering of Hainan Province Hainan University Haikou China; ^3^ Key Laboratory of Tropical Biological Resources of Ministry of Education and Hainan Engineering Research Center For Drug Screening and Evaluation Hainan University Haikou China; ^4^ Engineering Research Center For Hainan Biological Sample Resources of Major Diseases Key Laboratory of Emergency and Trauma of Ministry of Education The First Affiliated Hospital Hainan Medical University Haikou China; ^5^ School of Materials Science and Engineering Hainan University Haikou China

**Keywords:** bacterial pneumonia, magnetothermal therapy, mitophagy, nanozyme catalysis, nebulization delivery, sonodynamic therapy

## Abstract

Bacterial pneumonia treatment faces significant challenges due to uncontrolled inflammation and rising antibiotic resistance. Excessive inflammatory cytokine release and mitochondrial dysfunction exacerbate tissue damage, contributing to the disease's progression. Mitophagy, a process that eliminates dysfunctional mitochondria, helps regulate reactive oxygen species (ROS) levels and suppress inflammation. However, efficient bacteria clearance remains critical for effective inflammatory modulation. Traditional antibiotics struggle against resistant bacteria, necessitating alternative approaches. ROS‐based nanocatalytic therapies, particularly nanozymes, show potential for overcoming ROS generation limitations through external stimuli like ultrasound and magnetic fields. This study designs a sono‐magneto‐responsive nanozyme (BN@NF) composed of boron nanosheets (BN) and neodymium‐doped iron phosphide (Nd:Fe_2_P, NF), modified to form a Z‐scheme heterostructure. Delivered via nebulization, BN@NF generates ROS under combined internal and external stimuli, enabling efficient bacterial killing and biofilm disruption. It also modulates inflammation by inhibiting the NF‐κB pathway and promoting mitophagy, reducing pro‐inflammatory cytokine release. Additionally, BN@NF enhances macrophage polarization from the pro‐inflammatory M1 phenotype to the anti‐inflammatory M2 phenotype, aiding tissue repair. This approach provides a promising strategy for treating bacterial pneumonia by combining effective antibacterial action with intelligent anti‐inflammatory functions.

## Graphical abstract

Nebulized BN@NF nanozymes integrate boron nanosheets with Nd‐doped iron phosphide to form a sono‐magneto‐responsive Z‐scheme heterostructure. Under ultrasound and magnetic activation, BN@NF amplifies ROS generation, eradicates bacteria, disrupts biofilms, and reshapes pulmonary inflammation through NF‐κB suppression, mitophagy promotion, and M1‐to‐M2 macrophage repolarization, offering an integrated non‐antibiotic strategy for bacterial pneumonia therapy.

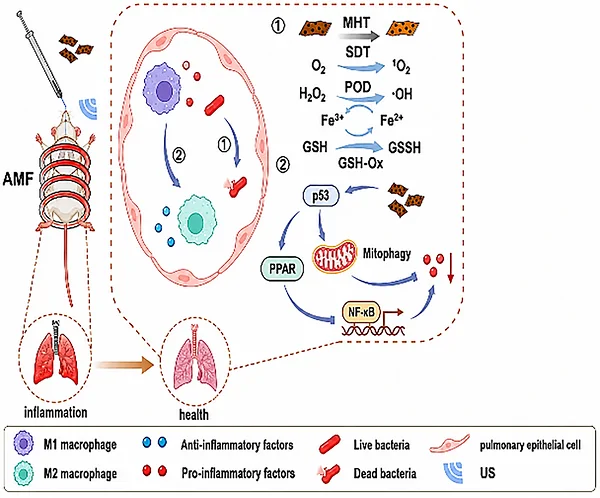

## 1 Introduction

Bacterial pneumonia remains a significant global health threat, complicated by two major issues: uncontrolled inflammatory responses and the rapid rise in antibiotic resistance [1, 2, 3]. The escalation of antibiotic resistance has led to a concerning increase in the mortality rates associated with bacterial infections, such as those caused by *Klebsiella pneumoniae*. While antibiotics are essential in combating bacterial infections, their development often fails to keep pace with evolving resistance mechanisms, making treatment increasingly ineffective [4, 5, 6, 7]. On the other hand, the inflammatory response to infection, especially excessive cytokine release, exacerbates tissue damage, further complicating the disease's management [4]. This inflammatory cascade, driven by mitochondrial dysfunction and oxidative stress, triggers the activation of nuclear factor kappa B (NF‐κB), which is a critical regulator of pro‐inflammatory cytokine release, such as tumor necrosis factor‐α (TNF‐α) and interleukin‐6 (IL‐6) [8, 9, 10, 11]. Furthermore, an imbalance in macrophage polarization, marked by excessive activation of the pro‐inflammatory M1 phenotype, further amplifies the inflammatory response. Mitophagy, a cellular quality control mechanism responsible for the selective degradation of damaged mitochondria [12, 13, 14], plays a crucial role in maintaining cellular homeostasis [15, 16, 17]. By eliminating dysfunctional mitochondria, mitophagy not only regulates reactive oxygen species (ROS) levels but also suppresses the amplification of inflammatory signaling [18, 19, 20, 21, 22, 23, 24], which may offer a strategy to mitigate the inflammatory microenvironment in bacterial pneumonia.

Efficiently addressing the inflammatory microenvironment in bacterial pneumonia requires a concurrent approach to bacterial clearance. Antibiotic resistance has significantly hindered the clinical management of infections, necessitating the exploration of alternative therapies. ROS‐based nanocatalytic therapy, which utilizes nanozymes capable of generating ROS in response to endogenous and exogenous stimuli, has shown promise in overcoming the limitations of traditional antibiotics [25]. However, the clinical transformation of ROS‐based therapies faces challenges due to kinetic bottlenecks in ROS generation, which are crucial for pathogen eradication [26, 27]. Nanozymes, with their ability to catalyze cascade reactions [28], can overcome these limitations through structural optimization and external physical field modulation, such as ultrasound (US) and magnetic fields [29, 30, 31, 32, 33, 34, 35]. Sonodynamic therapy (SDT), which uses US to activate sonosensitizers for ROS generation [36], holds particular promise for treating deep‐seated infections like pneumonia. However, the efficacy of SDT is often limited by rapid electron–hole pair recombination, reducing its antibacterial effectiveness [37, 38, 39, 40, 41, 42]. Meanwhile, magnetic hyperthermia therapy (MHT) employs alternating magnetic fields (AMF) to induce heat generation, promoting bacterial killing but with potential collateral damage due to non‐specific thermal effects [43, 44, 45, 46, 47]. Recent studies have reported inhalable MHT/SDT‐based platforms for bacterial lung infections and ultrasound‐switchable nanozymes for enhancing SDT against multidrug‐resistant bacterial infections, indicating that individual combinations such as SDT+MHT or nanozyme+SDT have been previously explored [48, 49]. However, the combination of these ultrasound‐triggered SDT and magnetic field‐triggered MHT to enhance nanozyme activity remains insufficiently investigated. More importantly, previous studies have mainly focused on pathogen elimination, whereas the spatiotemporal coordination between ROS‐mediated bacterial eradication and mitophagy‐mediated inflammation resolution has not been systematically investigated [49, 50, 51, 52, 53, 54, 55]. Therefore, there is an urgent need for a platform that integrates ROS generation with anti‐inflammatory modulation for effective treatment of bacterial pneumonia.

**SCHEME 1:**
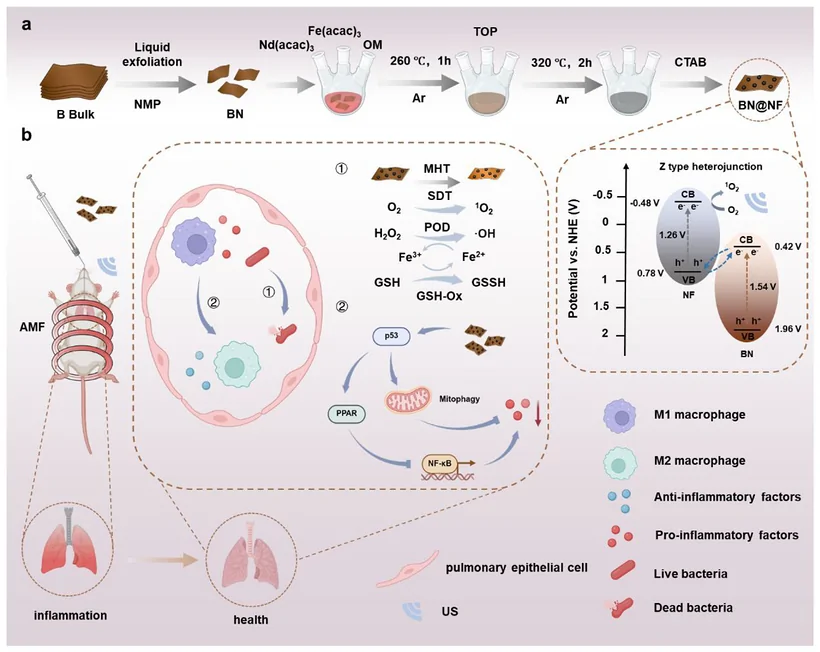
Schematic illustration of the preparation of BN@NF nanozyme (a) and its mechanism for antibacterial and anti‐inflammatory action in bacterial pneumonia therapy (b).

In this study, we present a strategy that integrates US and magnetic field‐responsive nanozymes (BN@NF) for the treatment of bacterial pneumonia (Scheme 1). The nanozyme, consisting of boron nanosheets (BN) and neodymium‐doped iron phosphide (Nd:Fe_2_P, NF) to form a Z‐scheme heterostructure, is modified with cetyltrimethylammonium bromide (CTAB) for enhanced stability and facilitated pulmonary delivery via nebulization, with the version synthesized without CTAB recorded as BNF for comparison. Upon administration, BN@NF generates ROS in response to both the infectious microenvironment and external US/magnetic stimuli. This simultaneous activation not only promotes bacterial killing but also disrupts biofilms, which are major barriers to effective infection treatment. In addition to its antimicrobial properties, BN@NF plays a pivotal role in modulating inflammation by inhibiting the NF‐κB pathway and promoting mitophagy. Fe^2+^ ions released in the acidic environment further enhance the depletion of protective glutathione (GSH) within bacteria, leading to increased ROS production and improved bacterial clearance. Moreover, BN@NF encourages macrophage polarization from the M1 pro‐inflammatory phenotype to the M2 anti‐inflammatory phenotype, which helps resolve inflammation and accelerates tissue repair [12, 18, 19, 56]. This study presents a promising approach for the design of ROS‐based antibacterial nanozymes, offering an effective strategy to tackle both the microbial and inflammatory challenges in bacterial pneumonia treatment.

## 2 Materials and Methods

### 2.1 Materials

High‐purity boron powder (99.9%, 10–20 µm), N‐methylpyrrolidone (NMP, 98%), and GSH were obtained from Macklin Reagent Co., Ltd. (Shanghai, China). Iron(III) acetylacetonate [Fe(acac)_3_], neodymium(III) acetylacetonate [Nd(acac)_3_], oleylamine (OM), trioctylphosphine (TOP, 95%), 3,3′,5,5′‐tetramethylbenzidine (TMB, 98%), 1,3‐diphenylisobenzofuran (DPBF, 97%), 5,5′‐dithiobis(2‐nitrobenzoic acid) (DTNB), 5,5‐dimethyl‐1‐pyrroline N‐oxide (DMPO), 2,2,6,6‐tetramethylpiperidine (TEMP), and 3‐(4,5‐dimethylthiazol‐2‐yl)‐2,5‐diphenyl tetrazolium bromide (MTT) were acquired from Adamas Reagent Co., Ltd. (Shanghai, China). Dimethyl sulfoxide (DMSO), cyclohexane, and anhydrous ethanol were supplied by Xilong Chemical Co., Ltd. (Guangdong, China). Dulbecco's Modified Eagle Medium (DMEM, high glucose), fetal bovine serum (FBS), and 0.25% trypsin‐EDTA were purchased from Gibco (Thermo Fisher Scientific, USA). Phosphate‐buffered saline (PBS), DAPI staining solution and Mitochondrial Membrane Potential Assay Kit with JC‐1 (M8650) were provided by Beijing Solarbio Science & Technology Co., Ltd. (Beijing, China). MitoTracker Green FM and LysoTracker Red DND‐99 were procured from Beyotime Biotechnology (Shanghai, China). Enzyme‐linked immunosorbent assay (ELISA) kits for detecting TNF‐α, Interleukin (IL)‐17, IL‐10, and IL‐1β were purchased from Multi Sciences Co., Ltd. (Hangzhou, China). Rabbit monoclonal antibodies targeting p53, p62/SQSTM1, LC3, PPARγ, Parkin, NF‐κB, as well as surface markers F4/80, CD86, and CD206, were supplied by Bioss Antibodies Inc. (Beijing, China). Horseradish peroxidase (HRP)‐conjugated goat anti‐rabbit IgG secondary antibody was obtained from Maxin Biotechnology Co., Ltd. (Fuzhou, China).

### 2.2 Characterization

The microstructure of BN@NF was analyzed using field emission scanning electron microscopy (FE‐SEM; Hitachi S4800, Japan) equipped with energy‐dispersive X‐ray spectroscopy (EDS; Super‐X, USA). The crystalline structure was examined via x‐ray diffraction (XRD; Haoyuan DX‐2700BH). Fourier transform infrared (FTIR) spectra were recorded on an iS50 spectrophotometer (Thermo Scientific, USA). Dynamic light scattering (DLS) and zeta potential measurements were performed using a Malvern Zetasizer Nano ZSE instrument. Elemental composition and chemical states were analyzed using x‐ray photoelectron spectroscopy (XPS; Axis Supra, UK). UV–Vis diffuse reflectance spectra (UV–Vis DRS) were acquired with a Thermo Fisher Evolution 220 spectrophotometer. Mott–Schottky analysis was performed on an electrochemical workstation (CHI660E, China) to assess the semiconductor properties of the nanomaterials. The detection of ROS was carried out using an X‐band (9.4 GHz) electron spin resonance (ESR) spectrometer (Bruker ESR5000, Germany).

### 2.3 Preparation of BN@NF

Preparation of BN: High‐purity boron powder (200 mg) was dispersed in 200 mL of NMP. The mixture was subjected to ultrasonic exfoliation for 6 h at room temperature using a cell disruptor (power: 1200 W; pulse duration: 2 s on, 2 s off). Subsequently, the dispersion was treated ultrasonically at 4°C for 24 h. After sonication, the mixture was centrifuged at 5000 rpm for 5 min to collect the supernatant. This supernatant was further centrifuged at 11 000 rpm for 30 min, and the resulting pellet was retained, while the supernatant was discarded. The precipitate was washed twice with ultrapure water via centrifugation at 11 000 rpm for 30 min each time. Finally, the product was pre‐frozen at −80°C for 3 h and lyophilized for 12 h to obtain BN.

Synthesis of Nd‐doped Fe_2_P (NF) nanoparticles: A series of NF nanoparticles were synthesized via a solvothermal method. Fe(acac)_3_ (denoted as x) and Nd(acac)_3_ (denoted as y) were introduced at molar ratios of x:y = 0.995:0.005 (0.5%), 0.99:0.01 (1%), 0.985:0.015 (1.5%), and 0.98:0.02 (2%), with corresponding masses of 351.4 mg/2.2 mg, 349.6 mg/4.4 mg, 347.9 mg/6.6 mg, and 346.1 mg/8.8 mg, respectively, into 50 mL OM. All samples were gradually heated to 260°C under an argon atmosphere and maintained for 1 h, followed by rapid injection of 3.3 mL TOP. The temperature was then increased to 320°C and held for 2 h. Upon cooling to room temperature, the products were separated by centrifugation at 12 000 rpm for 30 min. The supernatant was discarded, and the obtained solids were washed three times successively with ethanol and cyclohexane, then dried in a vacuum oven at 60°C. As a control, undoped Fe_2_P nanoparticles were synthesized using a similar method by adding 353.2 mg Fe(acac)_3_ (1.0 mmol) alone into 50 mL OM. The subsequent reaction, separation, and drying procedures were identical to those described above for the doped samples.

Preparation of BN@NF composites: BN@NF composites were fabricated via a stepwise method. First, ultrasonically exfoliated BN was dispersed in 50 mL of OM at amounts of 5.4 mg (0.5 mmol), 10.8 mg (1 mmol), 21.6 mg (2 mmol), and 43.2 mg (4 mmol), respectively. After stirring at room temperature for 2 h, 349.6 mg Fe(acac)_3_ (0.99 mmol) and 4.4 mg Nd(acac)_3_ (0.01 mmol) (NF precursor) were added to each group. Under argon protection, the mixture was heated to 260°C and maintained for 1 h, followed by rapid injection of 3.3 mL TOP. The temperature was then increased to 320°C and held for 2 h. After cooling to room temperature, the crude products were collected by centrifugation at 12 000 rpm for 30 min. The supernatant was discarded, and the solids were redispersed in cyclohexane. Under ultrasonication, the dispersion was slowly added to an ethanol solution containing cetyltrimethylammonium bromide (CTAB). After 10 min of ultrasonication, the mixture was shaken overnight. Finally, the products, BN@NF, were separated by centrifugation, washed three times with deionized water, and freeze‐dried. The obtained products exhibited BN to NF molar ratios of 1:2, 1:1, 2:1, and 4:1, respectively. For comparison, those synthesized using the same steps but without CTAB processing are recorded as BNF.

### 2.4 Magnetic Hyperthermia Testing

The magnetic hyperthermia heating performance of BN@NF was evaluated at different concentrations and AMF intensities. Centrifuge tubes containing 1 mL of BN@NF aqueous solutions at various concentrations were placed in an AMF. The real‐time temperature was measured using an infrared thermal imager (FLUKE TiS75). Temperature readings were recorded every 30 s for 10 min after applying an AMF at 498 kHz, with field intensities of 1.6, 1.2, and 0.8 mT. For testing magnetic hyperthermia cycle stability, the magnetic field was applied for 10 min, followed by a 10‐min pause. This cycle was repeated five times, and the temperature changes were recorded. The specific absorption rate (SAR) of BN@NF was also calculated to evaluate its magnetothermal conversion efficiency under AMF:

(1) $$SAR=\frac{dT}{dt}\times C_{p}\times \frac{m_{s}}{m_{n}}$$

In this equation, SAR represents the specific absorption rate (in W/g), m_s_ is the mass of the solution, m_n_ is the mass of the solute, and d_T_/d_t_ refers to the initial slope of the heating curve. This formula was used to compute SAR based on experimental heating data.

### 2.5 Sonodynamic Activity Testing

To assess the sonodynamic performance of BN@NF, 1 mL of BN@NF aqueous solution (50 µg/mL) and 1 mL of DPBF DMSO solution (25 µg/mL) were mixed under light‐protected conditions. The mixture was subjected to US stimulation (1.0 MHz, 50% duty cycle, 1.0 W/cm^2^) for different durations using a handheld therapeutic US device at 25°C and 42°C, respectively. The degradation of DPBF under various US irradiation times was monitored by measuring the UV–Vis absorption spectra with a UV–Vis spectrophotometer.

### 2.6 Peroxidase (POD)‐Like and Glutathione Oxidase (GSHOx)‐Like Enzyme Activity Assay

The POD‐like activity of BN@NF was evaluated under various conditions. 100 µL of BN@NF solution at different concentrations was mixed with 840 µL of acetate buffer at pH 5.4, 6.5, or 7.4. Then, 10 µL of TMB solution (20 mg/mL in DMSO) and 50 µL of H_2_O_2_ at varying concentrations were added. After thorough mixing, absorbance at 652 nm was measured at multiple time intervals using a full‐wavelength microplate reader. For evaluating magnetically enhanced enzymatic activity, the mixed solution was placed in the magnetic hyperthermia device and subjected to AMF (1.2 mT) for 10 min before measuring absorbance.

50 µL of BN@NF aqueous solution at different concentrations and 50 µL of GSH aqueous solution at different concentrations were added to a centrifuge tube containing 900 µL of buffer (pH 5.4, 6.5, or 7.4). After uniform mixing and incubation for the specified time, the mixture was centrifuged at 9000 rpm for 5 min. The supernatant was mixed with the prepared DTNB solution (4 mg/mL in DMSO) at a ratio of 19:1 (v/v), and the UV–Vis absorption spectrum of the mixture was recorded. For evaluating magnetically enhanced enzymatic activity, after mixing the BN@NF and GSH solutions, the mixture was placed in the magnetic hyperthermia device and subjected to AMF (1.2 mT) for 10 min before proceeding with the DTNB reaction steps.

### 2.7 ESR Detection of Hydroxyl Radicals (•OH) and Singlet Oxygen (^1^O_2_)

The generation of ^1^O_2_ by BN@NF under US and the production of ·OH by POD‐like enzyme in BN@NF were monitored by ESR. Briefly, a well‐dispersed BN@NF suspension at the indicated concentration was mixed with the corresponding ROS trapping agent. The reaction was initiated either by US irradiation (1.0 MHz, 50% duty cycle, 1.0 W/cm^2^) or by the addition of H_2_O_2_ to a final concentration of 1.0 mM. DMPO and TEMP were used as spin‐trapping agents for ·OH and ^1^O_2_, respectively. The resulting solution was transferred into a capillary tube and placed in the ESR spectrometer for measurement. For assays designed to evaluate magnetic enhancement, a temperature‐controlled simulation mimicking magnetic hyperthermia conditions was used instead of direct AMF exposure during ESR measurement.

### 2.8 Bacterial Characterization

#### 2.8.1 Bacterial Culture

Single colonies of the multidrug‐resistant strain *Klebsiella pneumoniae* (ATCC BAA‐2472, designated MDR‐*K.p*) and the methicillin‐resistant *Staphylococcus aureus* (ATCC 43300, designated MRSA) were selected from agar plates and separately inoculated into LB broth, then incubated overnight at 37°C with shaking. The primary cultures were inoculated into fresh LB medium at a ratio of 1:100 and subcultured at 37°C for approximately 6 h. Once the optical density (OD) at 600 nm (OD_600_) reached 0.5, corresponding to a bacterial density of 10^8^ CFU/mL, the secondary culture was harvested for further experiments.

#### 2.8.2 Determination of Antibacterial Activity

The antibacterial activity of BN@NF was evaluated using the plate coating method. Bacterial suspensions and BN@NF were adjusted to final concentrations of 10^7^ CFU/mL and 2 mg/mL, respectively. Experimental groups were established as follows: AMF+US, BN@NF, BN@NF+AMF, BN@NF+US, and BN@NF+AMF+US. The control group was PBS treatment group (Control). US denotes ultrasound irradiation (1.0 W/cm^2^, 50% duty cycle, 10 min), while AMF has the following parameters: 498 kHz, 1.2 mT, and a duration of 10 min. After the treated bacterial suspensions were spread on LB agar and incubated at 37°C for 12 h, the colonies were imaged and counted. The survival rate was calculated using Equation (2):

(2) $$\mathrm{Survival}\;\mathrm{rate}\;\left(\%\right)=\mathrm{CFUtreatment}/\mathrm{CFUPBS}\times 100\%$$

where CFU_treatment_ and CFU_PBS_ represent the bacterial colony counts in the treatment and PBS control groups, respectively. Data are presented as the mean of three independent replicates.

#### 2.8.3 Bacterial Morphology Observation

After treatment, bacterial samples were rinsed three times with physiological saline, centrifuged, and fixed overnight at 4°C using 2.5% glutaraldehyde. The specimens were dehydrated through a graded ethanol series, spending 5 min at each concentration. The morphology and integrity of the bacteria were observed via SEM to assess the antibacterial mechanism of BN@NF.

#### 2.8.4 Bacterial Cytoplasmic Leakage Assay

To evaluate the release of bacterial DNA and proteins under different treatment conditions, the treated samples were subjected to centrifugation at 6000 rpm for 5 min at 4°C. The resulting supernatant was collected for analysis. Absorbance readings at 260 nm (indicating nucleic acids) and 280 nm (indicating proteins) were measured using an ultra‐microvolume UV–Vis NanoDrop 2000C Spectrophotometer (Thermo Scientific NanoDrop Products).

#### 2.8.5 Bacterial Live/Dead Fluorescent Staining

Bacteria from different treatment groups were rinsed three times with physiological saline and collected by centrifugation. The samples from each group were incubated with SYTO‐9 and propidium iodide (PI) in the dark for 20 min using the Live/Dead BacLight Bacterial Viability Kit (Thermo Fisher Scientific, Inc.). The mixture was then washed three times with physiological saline. Next, 100 µL of the stained bacterial solution was transferred to a glass coverslip for observation and photography under an inverted fluorescence microscope (DMi8, Leica, Germany). The fluorescence intensities of SYTO‐9 and PI were quantitatively analyzed. All experiments were conducted in triplicate.

### 2.9 Bacterial Biofilm Evaluation

#### 2.9.1 Bacterial Biofilm Cultivation

Cell climbing slides (9 mm in diameter) were placed into a 24‐well plate. Each well was inoculated with 500 µL of bacterial suspension at a concentration of 10^8^ CFU/mL and incubated at 37°C for 72 h to allow biofilm formation. The culture medium was refreshed with sterile LB broth every 24 h. After incubation, the slides were rinsed gently three times with PBS to remove non‐adherent bacteria, leaving behind mature, dense biofilms on the slides for subsequent experimental procedures.

#### 2.9.2 Biofilm Live/Dead Staining

Mature biofilms were subjected to treatments from the six experimental groups used in the antibacterial activity assay (Control, US+AMF, BN@NF, BN@NF+AMF, BN@NF+US, BN@NF+AMF+US). The control group was treated with PBS. US treatment was applied at an intensity of 1.0 W/cm^2^ with a 50% duty cycle for 10 min, and the AMF was set to 1.2 mT for 10 min. After treatment, the biofilms were rinsed three times with sterile physiological saline. The biofilms were then co‐stained with SYTO‐9 and PI under light‐protected conditions for 20 min, followed by three washes with saline. Three‐dimensional structural images of the stained biofilms were acquired by confocal laser scanning microscopy (CLSM). All experiments were conducted in triplicate.

#### 2.9.3 Biofilm Crystal Violet (CV) Staining and Quantification

Following the assigned treatments, mature biofilms were fixed by applying 500 µL of a 2.5% glutaraldehyde solution per well for 20 min. The fixed biofilms were then stained with 0.1% CV aqueous for 20 min. After staining, the samples were gently rinsed three times with sterile physiological saline. Once dried, images of the CV‐stained biofilms were taken. The stained biofilms were then subjected to dye elution with 200 µL of 33% glacial acetic acid per well. The OD of the resulting solution was measured at 570 nm, and the relative biofilm biomass was determined using Equation (3):

(3) $$\mathrm{Relative}\;\mathrm{biofilm}\;\mathrm{biomass}\left(\%\right)=\left(ODt/ODc\right)\times 100\%$$

where OD_t_ and OD_c_ represent the mean OD values of the treatment and PBS control groups, respectively. Data are derived from three independent replicates.

### 2.10 In Vitro Cellular Analysis

#### 2.10.1 Cell Culture

The mouse fibroblast cell line L929 and the monocyte macrophage cell line RAW264.7 were provided by Hunan Fenghui Biotechnology Co., Ltd. Both L929 cells and RAW264.7 cells were maintained in DMEM, supplemented with 10% FBS and 1% penicillin‐streptomycin. Cells were incubated under standard humidified conditions at 37°C with 5% CO_2_.

#### 2.10.2 Cytotoxicity Test

The cytotoxicity of BN@NF toward L929 cells was evaluated using the MTT assay at various concentrations. Briefly, cells were seeded in 96‐well plates at 1 × 10^4^ cells per well in 200 µL of DMEM and cultured for 24 h. Subsequently, the cells were exposed to BN@NF at concentrations ranging from 0 to 400 µg/mL for another 24 h. MTT was added to each well to achieve a final concentration of 500 µg/mL, and the plates were incubated at 37°C for 4 h in darkness. After aspirating the supernatant, 200 µL of DMSO was added to dissolve the formazan precipitates. The plates were shaken in the dark for 20 min, and absorbance was measured at 490 nm using a microplate reader. Cell viability was calculated based on the untreated control groups using Equation (4):

(4) $$\mathrm{Survival}\;\mathrm{rate}\left(\%\right)=\left(OD490sample/OD490control\right)\times 100\%$$

where OD490_control_ and OD490_sample_ represent the mean absorbance values of the untreated control and BN@NF‐treated sample groups, respectively. Data are obtained from three independent experiments.

#### 2.10.3 Hemolysis Assay

To evaluate the biocompatibility of BN@NF, erythrocyte hemolysis assays were conducted. Whole blood was collected from BALB/c mice using EDTA‐anticoagulated tubes, then centrifuged at 3000 rpm for 10 min to obtain red blood cells (RBCs). The RBC pellet was washed with PBS and reconstituted to a 10% (v/v) suspension in the same buffer. Gradient concentrations of BN@NF were applied to the RBC suspension, using PBS and deionized water as the negative and positive controls, respectively. After incubation at 37°C for 2 h, the samples were centrifuged again under identical conditions. The supernatant was photographed, and its absorbance was measured at 540 nm. The hemolysis percentage was determined using Equation (5):

(5) $$\begin{array}{ccc} \mathrm{Hemolysis}\;\mathrm{rate}\left(\%\right) & = & \frac{OD540_{eachgroup}}{OD540_{positivegroup}-OD540_{negativegroup}}\\ & & \times 100\% \end{array}$$

where OD540_each group_, OD540_negative group_, and OD540_positive group_ represent the absorbance values of the experimental sample, negative control, and positive control supernatants measured at 540 nm, respectively. All experiments were conducted in triplicate.

#### 2.10.4 Detection of Mitochondrial Membrane Potential (ΔΨm)

The mitochondrial membrane potential (ΔΨm) of cells was measured using the JC‐1 probe. RAW264.7 cells were cultured in confocal dishes for 24 h, followed by incubation with different groups of samples for 6 h. After specific treatments, cells were incubated with 10 µg/mL JC‐1 for 15 min and subsequently visualized using a confocal laser scanning microscope.

#### 2.10.5 Staining and Visualization of Mitochondria and Lysosomes in Cells

Mitochondrial and lysosomal staining was performed using fluorescent probes, Mito‐Tracker Green and Lyso‐Tracker Red, respectively, to label these organelles within viable cells. RAW264.7 cells were cultured in confocal dishes for 24 h, then exposed to different experimental groups and incubated for another 24 h. After treatment, the prepared fluorescent probe working solution was added according to the manufacturer's instructions and incubated at 37°C in the dark for 30 min. After the incubation period, the staining solution was carefully removed, and the cells were washed three times with PBS. The fluorescence from the stained cells was visualized and captured using laser scanning confocal microscopy.

#### 2.10.6 Macrophage Polarization by the Flow Cytometry

The effect of BN@NF on macrophage polarization was evaluated by flow cytometry. RAW264.7 cells in the M0 state were first polarized toward the M1 phenotype by stimulation with lipopolysaccharide (LPS, 50 µg/mL) for 12 h. BN@NF was then added to the cells, which were further cultured under the specified experimental conditions. Control and AMF+US groups were treated with PBS. The positive control group was induced with interleukin‐4 (IL‐4). After 4 h of treatment, cells from each group were collected, labeled with specific markers, and analyzed by flow cytometry.

#### 2.10.7 Western Blotting

Protein expression was analyzed by Western blotting. After treatment, RAW264.7 cells were harvested and lysed on ice using RIPA buffer supplemented with protease inhibitor phenylmethylsulfonyl fluoride (PMSF) to prevent protein degradation. The lysates were centrifuged at 12 000 × g for 10 min at 4°C, and the supernatant was collected for protein quantification using a BCA assay. Equal amounts of protein were denatured in loading buffer by heating at 100°C for 5 min. Proteins were separated by SDS‐PAGE and transferred to a PVDF membrane. After blocking for 1 h, the membranes were incubated with specific primary antibodies at 4°C overnight, followed by incubation with corresponding HRP‐conjugated secondary antibodies for 1 h at room temperature. Signal detection was performed using enhanced chemiluminescence (ECL) substrate, and images were acquired in the dark.

### 2.11 Evaluation of BN@NF Biodistribution in Vivo

Indocyanine green (ICG) dye was mixed with BN@NF nanoparticles at a mass ratio of 1:1, sonicated for 30 min, and then incubated overnight at 37°C on a shaker. After incubation, the mixture was centrifuged at 5000 rpm and washed three times with water. The collected product was subsequently lyophilized to obtain ICG‐labeled BN@NF nanoparticles. To reduce imaging interference caused by abdominal hair, the mice were shaved before treatment. The mice were then administered ICG‐labeled BN@NF by nebulized inhalation. Fluorescence signals were recorded in vivo and ex vivo using a full‐spectrum deep bio‐imaging system (Biolight AniView Phoenix 600SE, China). For ex vivo analysis, the heart, liver, spleen, lungs, and kidneys were collected and imaged. The fluorescence intensity of each organ was then quantified using ImageJ software (NIH) to evaluate the biodistribution of BN@NF.

### 2.12 Treatment of Established BALB/c Mouse Pneumonia Model

Male BALB/c mice (6–8 weeks old) were purchased from the Hunan Provincial Experimental Animal Center. All mice were monitored twice daily for pain, distress, or moribund status, and animals showing loss of > 20% initial body weight within 24 h, persistent anorexia, inability to access food or water, severe lethargy, hypothermia (< 35°C), labored breathing, seizures, or paralysis were immediately euthanized by cervical dislocation. No mice reached these humane endpoint criteria (HECs) before the scheduled 3 d endpoint approved by the Animal Ethics Committee of Hainan University (Approval No. HNUAUCC‐2025‐00466). Anesthesia was induced by intraperitoneal injection of 0.3% (v/v) sodium pentobarbital at a dosage of 50 mg/kg. Each mouse was administered 50 µL of MDR‐*K.p* bacterial suspension (1 × 10^9^ CFU/mL) via pulmonary nebulization using a dedicated small animal nebulizer. Twenty‐four hours post‐infection, mice were randomly assigned to seven groups (n = 6 per group): Healthy (uninfected, untreated), Control (infected, untreated), AMF+US, BN@NF, BN@NF+AMF, BN@NF+US, and BN@NF+AMF+US. US parameters were set as follows: intensity 1.0 W/cm^2^, duty cycle 50%, and duration 10 min. AMF conditions were set at 498 kHz, 1.2 mT, for 10 min. At the end of the 3‐day experimental period, the mice were euthanized, and major organs (heart, liver, spleen, lungs, and kidneys) were harvested and immersion‐fixed in a 4% paraformaldehyde solution for sectioning and staining. Lung tissues were homogenized, and residual bacterial count was quantified via the plate coating method. Blood samples were collected for hematological analysis, and serum was assessed for biochemical markers. Inflammatory cytokine concentrations in both bronchoalveolar lavage fluid (BALF) and serum samples were determined using commercially available ELISA kits.

### 2.13 Anti‐Inflammatory Mechanisms by in Vivo Transcriptomic Analysis

Transcriptomic analysis was performed on lung tissue samples obtained from both control and BN@NF+AMF+US‐treated mice. Lung samples were dissected, rinsed in diethypyrocarbonate (DEPC)‐treated water to remove blood, blotted dry, and flash‐frozen in liquid nitrogen. RNA was extracted, enriched, and purified. The purified mRNA was fragmented, and cDNA libraries were constructed through reverse transcription with random primers, followed by sequencing. Raw sequencing data were quality‐controlled and filtered using fastp. Gene expression quantification and differential analysis were performed. Functional enrichment analysis was subsequently conducted.

### 2.14 Statistical Analysis

The statistical significance of intergroup differences was assessed using one‐way analysis of variance (ANOVA). The following thresholds for significance were used: *p < 0.05, **p < 0.01, ***p < 0.001, and ****p < 0.0001; “ns” denotes not significant. All statistical analyses were conducted using GraphPad Prism (version 8.0, GraphPad Software, United States).

## 3 Results and Discussion

### 3.1 Synthesis and Characterization of BN@NF

Nd doping in Fe_2_P was first optimized because a strong AMF response was required for subsequent catalytic activation. A series of Nd‐doped iron phosphides with doping levels from 0.5% to 2% was therefore prepared and screened. As shown in Figure S1a, the diffraction patterns changed with increasing Nd content, and a gradual evolution from Fe‐related impurity peaks toward Fe_3_O_4_‐related reflections was observed. Among these samples, the diffraction profile of the 1% Nd‐doped product matched the standard Fe_2_P phase most closely, which indicated that the Fe_2_P crystal framework was largely preserved at this doping level. The AMF heating profiles further showed that all Nd‐doped samples displayed higher heating efficiency than pristine Fe_2_P (43.4°C after 10 min), whereas the 1% Nd‐doped sample showed the strongest response and reached approximately 89°C within 10 min (Figure S1b). This result indicated that moderate Nd incorporation effectively improved magnetic‐to‐thermal energy conversion. The enhanced magnetothermal behavior was attributed to the regulation of magnetic anisotropy and exchange coupling by Nd^3+^ doping. Rare‐earth ions with unquenched 4f orbital angular momentum were reported to introduce strong single‐ion anisotropy, thereby increasing the magnetocrystalline anisotropy energy and the effective anisotropy field of Fe‐based materials, which in turn promoted hysteresis loss under AMF exposure [57]. In parallel, partial substitution of Fe lattice sites by rare‐earth ions was reported to alter the lattice constant ratio and strengthen long‐range magnetic coupling among adjacent Fe atoms, which further improved magnetic ordering and heating efficiency [58]. However, the dependence of heating performance on Nd content was not monotonic. Instead, the optimal effect was obtained at 1% doping. This behavior was likely caused by a balance between two competing factors. At moderate doping, effective anisotropy pinning centers were introduced, the hysteresis loop area was enlarged, and magnetic relaxation was better synchronized with the applied AMF [57]. In contrast, excessive doping might have disrupted the continuity of the exchange‐coupling network in the Fe_2_P matrix or shifted the Curie temperature away from the practical operating range, which would have reduced heating efficiency [58, 59, 60, 61]. Accordingly, the 1% Nd‐doped material was selected for subsequent study and was denoted as NF.

The BN@NF composite was then constructed by combining BN with the optimized NF component. BN was first obtained by ultrasonic liquid‐phase exfoliation and was used as a template for subsequent growth. NF nanoparticles were then grown in situ on the BN surface at 320°C through thermal decomposition using Fe(acac)_3_ and Nd(acac)_3_ as metal precursors and trioctylphosphine (TOP) as the phosphorus source. The directly freeze‐dried product was denoted as BNF, whereas CTAB modification yielded the final dispersible composite, BN@NF. To optimize the composition, materials with BN:NF mass ratios of 1:2, 1:1, 2:1, and 4:1 were prepared. XRD analysis showed that both BN‐ and Fe_2_P‐related structural features were retained in all composites, whereas the relative contribution of BN gradually increased with increasing BN content (Figure S1c). Under AMF, the heating capacity decreased as the BN fraction increased (Figure S1d). After 10 min, the temperatures of the 1:2, 1:1, 2:1, and 4:1 groups reached approximately 57.8°C, 49.7°C, 47.3°C, and 37.9°C, respectively. Catalytic ROS generation was then preliminarily evaluated by monitoring DPBF consumption. As shown in Figure S1e, the ROS‐generating performance did not follow the same trend as the heating behavior. Instead, DPBF degradation first increased and then decreased with increasing BN content, and the strongest response was obtained at the BN:NF ratio of 2:1. After 10 min, the A_t_/A_0_ value at 414 nm decreased to approximately 0.42 in the 2:1 group, whereas higher residual values were observed for the 1:2, 1:1, and 4:1 groups. These results indicated that ROS generation was not governed by magnetothermal output alone, but was also strongly influenced by the interfacial interaction between BN and NF. Therefore, the 2:1 formulation was selected as the final material because it provided a more balanced combination of magnetic heating and catalytic ROS production.

**FIGURE 1:**
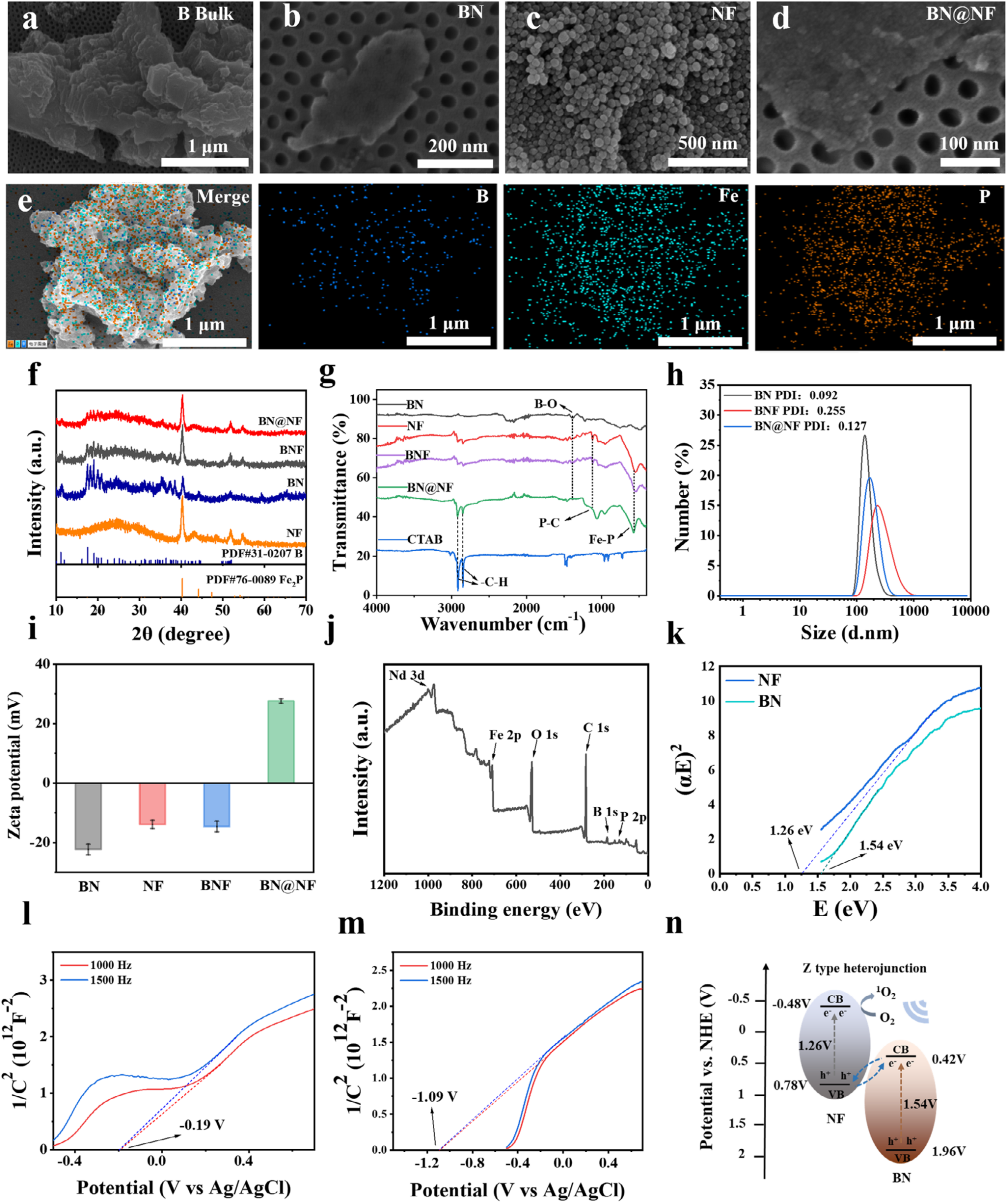
Characterization of BN@NF. SEM images of (a) bulk boron (B Bulk), (b) BN, (c) neodymium‐doped iron phosphide (NF), and (d) BN@NF. (e) EDS elemental mapping of BN@NF. (f) XRD patterns and (g) FTIR spectra of BN, NF, BNF, and BN@NF. (h) DLS size distributions of different samples. (i) Zeta potential measurements of BN, NF, BNF, and BN@NF (n = 3). (j) XPS survey spectra of BN@NF. (k) UV–Vis DRS of BN and NF. Mott–Schottky plots of (l) BN and (m) NF. (n) Schematic band alignment diagram of BN and NF.

The morphology of each component and of the final hybrid was then examined by SEM. Bulk boron displayed a stacked block‐like morphology at the micrometer scale (Figure 1a), whereas exfoliated BN showed a much thinner nanosheet‐like structure (Figure 1b), indicating that effective delamination had been achieved. NF was composed of densely packed spherical nanoparticles with a diameter of about 20 nm (Figure 1c). After assembly, these nanoparticles were found to be distributed on the BN surface, and a rough sheet‐supported granular architecture was observed in BN@NF (Figure 1d). EDS elemental mapping further showed that B, Fe, and P were uniformly distributed throughout the BN@NF structure (Figure 1e), confirming that the NF nanoparticles were successfully integrated with the BN nanosheets rather than being merely phase‐separated aggregates. This intimate interfacial contact was considered important for facilitating electron migration and maximizing the exposure of catalytically active sites. The phase composition and surface chemistry of BN@NF were further analyzed by XRD and FTIR. In Figure 1f, BN@NF displayed the characteristic diffraction peaks of both BN and Fe_2_P, which were consistent with those of BNF and confirmed successful composite formation. The peak at 2θ ≈ 17.6° was assigned to the (104) plane of α‐rhombohedral boron (PDF#31‐0207) [62], whereas the reflection at 2θ ≈ 40.3° was assigned to the (111) plane of hexagonal Fe_2_P (PDF#76‐0089) [63]. No major structural collapse was detected after assembly or CTAB treatment. The FTIR spectra provided additional evidence for successful integration (Figure 1g). The broad absorption in the range of 1300–1500 cm^−1^ was assigned to B‐O vibration [64], the band near 576 cm^−1^ was attributed to Fe‐P vibration [65], and the band at 1100–1200 cm^−1^ was associated with P‐C vibration from TOP‐derived surface species [66]. In addition, the absorption at 2800–3000 cm^−1^ was assigned to C‐H stretching from CTAB [67]. These results suggested that the final material retained the characteristic chemical signatures of both BN and NF and that the surface of the hybrid was successfully modified by CTAB.

The colloidal behavior of the hybrid system was also markedly improved after surface modification. The unmodified BNF intermediate appeared as aggregated clusters in the SEM image (Figure S2a), and obvious sedimentation was observed after 120 h of standing, whereas BN@NF remained visibly dispersed over the same period (Figure S2b). Consistently, the hydrodynamic diameter of BN@NF was maintained within a relatively narrow range of about 150–180 nm over 120 h, while the PDI remained stable at about 0.13‐0.17 (Figure S2c,d). In the DLS profiles, the average hydrodynamic diameter increased from 142 nm for BN to 220 nm for BNF, which suggested partial aggregation after direct composite formation. After CTAB modification, the average size decreased to 164 nm, and the size distribution became narrower (Figure 1h). The corresponding PDI values also supported this trend, with BN, BNF, and BN@NF showing PDIs of 0.092, 0.255, and 0.127, respectively. Meanwhile, the zeta potential shifted from negative values for BN (−22.3 ± 1.8 mV), NF (−13.9 ± 1.3 mV), and BNF (−14.6 ± 1.8 mV) to a positive value for BN@NF (27.6 ± 0.8 mV) (Figure 1i). This charge reversal was attributed to CTAB‐assisted interfacial engineering and was consistent with the improved dispersion stability of the final nanocomposite.

The surface chemical states and electronic structure of BN@NF were then analyzed to clarify the basis of its catalytic behavior. The XPS survey spectrum confirmed the presence of Nd, Fe, O, C, B, and P in BN@NF (Figure 1j), which verified successful integration of all expected components. In the high‐resolution Fe 2p spectrum, Fe^2+^ and Fe^3+^ species were simultaneously detected together with their satellite peaks (Figure S3), indicating the coexistence of mixed‐valence iron states on the composite surface. This feature was considered beneficial for redox cycling during ROS generation. The optical and band structure analyses further suggested that a Z‐scheme heterostructure was formed between BN and NF. According to the UV–Vis DRS, the bandgaps of NF and BN were estimated to be 1.26 and 1.54 eV, respectively (Figure 1k). The Mott–Schottky plots showed flat‐band potentials of about −1.09 V for NF and −0.19 V for BN vs. Ag/AgCl (Figure 1l,m). After combination with the bandgap results, the band alignment shown in Figure 1n was established, in which the conduction band of NF remained more negative and the valence band of BN remained more positive. This Z‐scheme configuration was favorable because strong reduction and oxidation capacities were simultaneously preserved, thereby facilitating interfacial charge separation and ROS production under external stimulation.

### 3.2 Magnetothermal and Sonodynamic Performance of BN@NF

**FIGURE 2:**
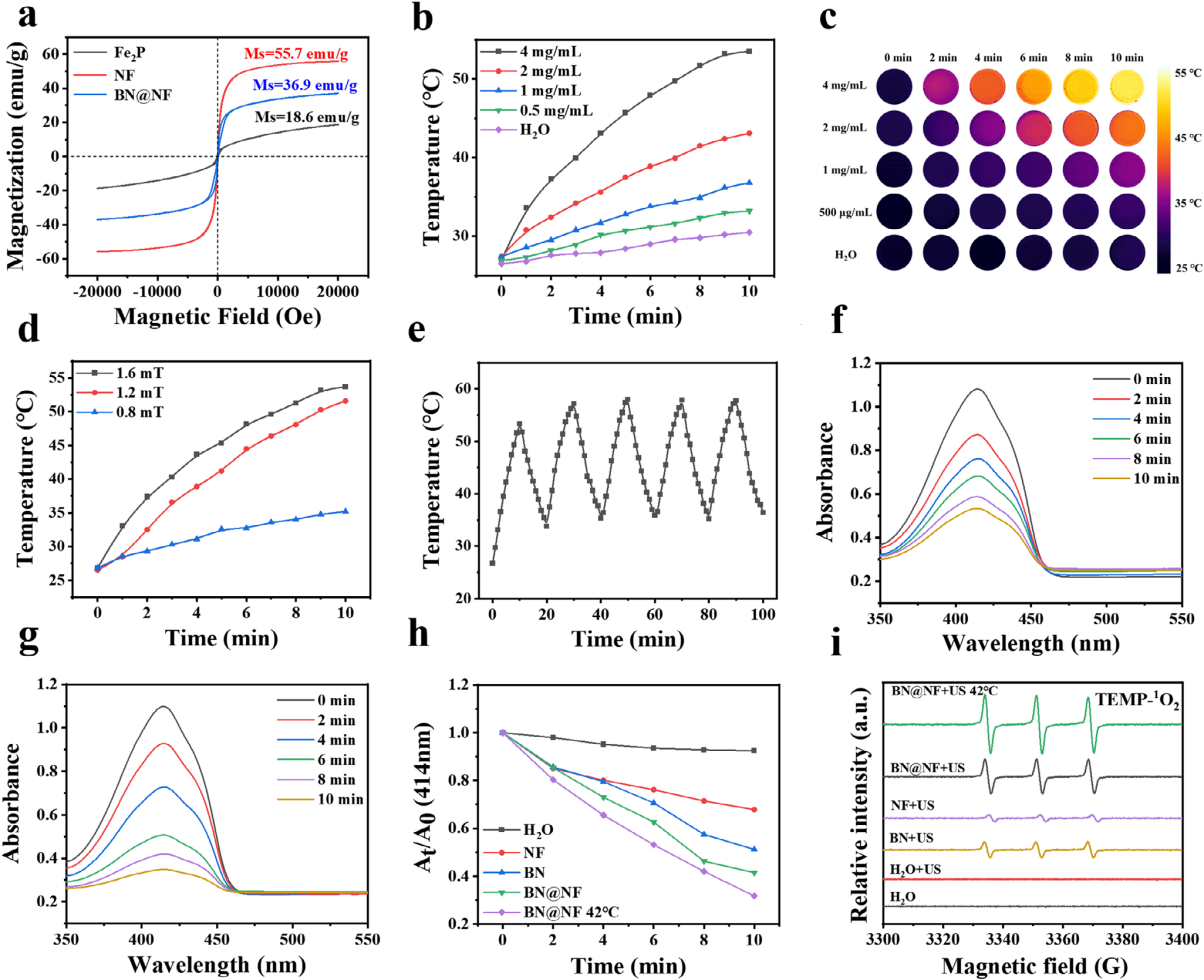
Magnetothermal and sonodynamic performance of BN@NF under in vitro conditions. (a) Magnetic hysteresis loops of Fe_2_P, NF, and BN@NF. (b) Temperature elevation curves of BN@NF under AMF at varying concentrations. (c) Real‐time infrared thermographic images of BN@NF during AMF exposure. (d) Magnetothermal heating profiles of BN@NF under different magnetic field intensities. (e) Cyclic magnetothermal stability of BN@NF over five heating‐cooling cycles. Time‐dependent UV–Vis spectra of ^1^O_2_ generation under US (1.0 MHz, 1.0 W/cm^2^) from BN@NF at (f) 25°C and (g) 42°C, respectively. (h) Comparative DPBF degradation rates of water (H_2_O), NF, BN, BN@NF, and temperature‐controlled BN@NF (42°C). (i) ESR spectra detecting ^1^O_2_ generation under US irradiation for H_2_O (control), NF, BN, BN@NF, and BN@NF at 42°C.

A rapid magnetic response of the BN@NF aqueous dispersion was first observed under a static magnetic field, as evidenced by its obvious migration and aggregation toward the magnet within 30 s (Figure S4). This result indicated that sufficient magnetic responsiveness was retained after hybridization. The magnetic properties of Fe_2_P, NF, and BN@NF were then characterized by vibrating sample magnetometry (Figure 2a and Figure S5). Fe_2_P showed a saturation magnetization (M_s_) of 18.6 emu/g, whereas Nd‐doped NF showed a markedly increased M_s_ of 55.7 emu/g. After coupling with BN, BN@NF still maintained a relatively high M_s_ of 36.9 emu/g, although the value was reduced because of the introduction of the nonmagnetic BN component. A similar trend was observed for the coercivity (H_c_). The H_c_ values of Fe_2_P, NF, and BN@NF were measured to be 111.7, 11.6, and 51.6 Oe, respectively. These changes suggested that Nd incorporation and subsequent BN coupling altered the magnetic interactions and anisotropy of the Fe_2_P‐based system, while adequate field responsiveness was still preserved in BN@NF for magnetic actuation.

The magnetothermal behavior of BN@NF was then evaluated under an AMF. Under AMF exposure (1.6 mT, 496 kHz), a clear concentration‐dependent temperature increase was observed (Figure 2b,c). After 10 min, the temperatures of BN@NF dispersions at 0.5, 1, 2, and 4 mg/mL increased to 33.1°C, 36.7°C, 43.1°C, and 53.5°C, respectively, whereas water showed only a slight increase of approximately 4°C over the same period. The infrared thermographic images were consistent with these temperature elevation curves and further confirmed the efficient magnetic heating behavior of BN@NF. A magnetic field intensity‐dependent heating pattern was also observed (Figure 2d). After 10 min of AMF treatment, the temperatures reached 35.2°C at 0.8 mT, 51.6°C at 1.2 mT, and 53.5°C at 1.6 mT. These results showed that the heating output of BN@NF could be tuned by both concentration and magnetic field intensity. In addition, the SAR values of Fe_2_P, NF, and BN@NF were calculated to be 36.5, 157.5, and 109.9 W/g, respectively (Table S1). This trend was consistent with the measured M_s_ values, further indicating that Nd doping enhanced the magnetothermal performance of Fe_2_P, while the composite still preserved strong heat‐generation capability after BN integration. The repeatability of AMF‐triggered heating was further assessed by five consecutive heating‐cooling cycles. As shown in Figure 2e, no obvious attenuation in the peak temperature was detected during repeated on/off switching of the magnetic field, and the heating‐cooling profiles remained highly reproducible throughout the test period. This result indicated that BN@NF possessed stable magnetothermal conversion behavior under cyclic stimulation. Such stability was important for subsequent therapeutic applications, because repeated field exposure might be required during pulmonary intervention. Moreover, the ability of BN@NF to reach the mild hyperthermia range under controllable conditions provided a practical basis for evaluating whether heat could further amplify US‐triggered ROS production.

The sonodynamic performance of BN@NF was therefore investigated by monitoring the degradation of DPBF, a probe for ^1^O_2_, under US irradiation. In pure water, only negligible spectral changes were observed during US treatment (1.0 MHz, 1 W/cm^2^), indicating that ROS generation by US alone was limited under these conditions (Figure S6). By contrast, obvious time‐dependent DPBF degradation was observed in the presence of NF, BN, and BN@NF under US treatment, which indicated that all three materials contributed to sonodynamic ROS generation (Figure S7 and Figure 2f). Among them, the BN@NF group showed the strongest DPBF consumption at 25°C. More than 50% of the DPBF signal was lost within 10 min under US irradiation (1.0 MHz, 1.0 W/cm^2^), which supported the formation of an efficient heterostructure and improved interfacial charge separation. Because BN@NF also exhibited strong magnetothermal activity, its sonodynamic performance was further evaluated at 42°C. Under this condition, the DPBF signal was almost completely depleted within 10 min (Figure 2g), indicating that mild heating further amplified ^1^O_2_ production. This enhancement was further supported by the quantitative analysis of the normalized absorbance at 414 nm (Figure 2h). The BN@NF+US group at 42°C showed the lowest A_t_/A_0_ value among all groups, indicating the highest ^1^O_2_ generation efficiency. This result suggested that thermal input facilitated the sonodynamic process, likely by lowering the reaction barrier, accelerating charge transfer, and promoting the separation of electron–hole pairs. ESR analysis provided direct evidence for this conclusion. As shown in Figure 2i, the characteristic ^1^O_2_ signal was the strongest in the BN@NF+US group at 42°C, whereas much weaker signals were detected in the H_2_O, NF, BN, and BN@NF groups without thermal assistance. Taken together, these results demonstrated that BN@NF combined efficient magnetothermal conversion with enhanced sonodynamic ^1^O_2_ generation. More importantly, the sonodynamic output was markedly strengthened under mild hyperthermic conditions, which suggested that AMF‐assisted heating and US activation could act cooperatively to amplify ROS production.

### 3.3 POD‐Like and GSHOx‐Like Activities of BN@NF

**FIGURE 3:**
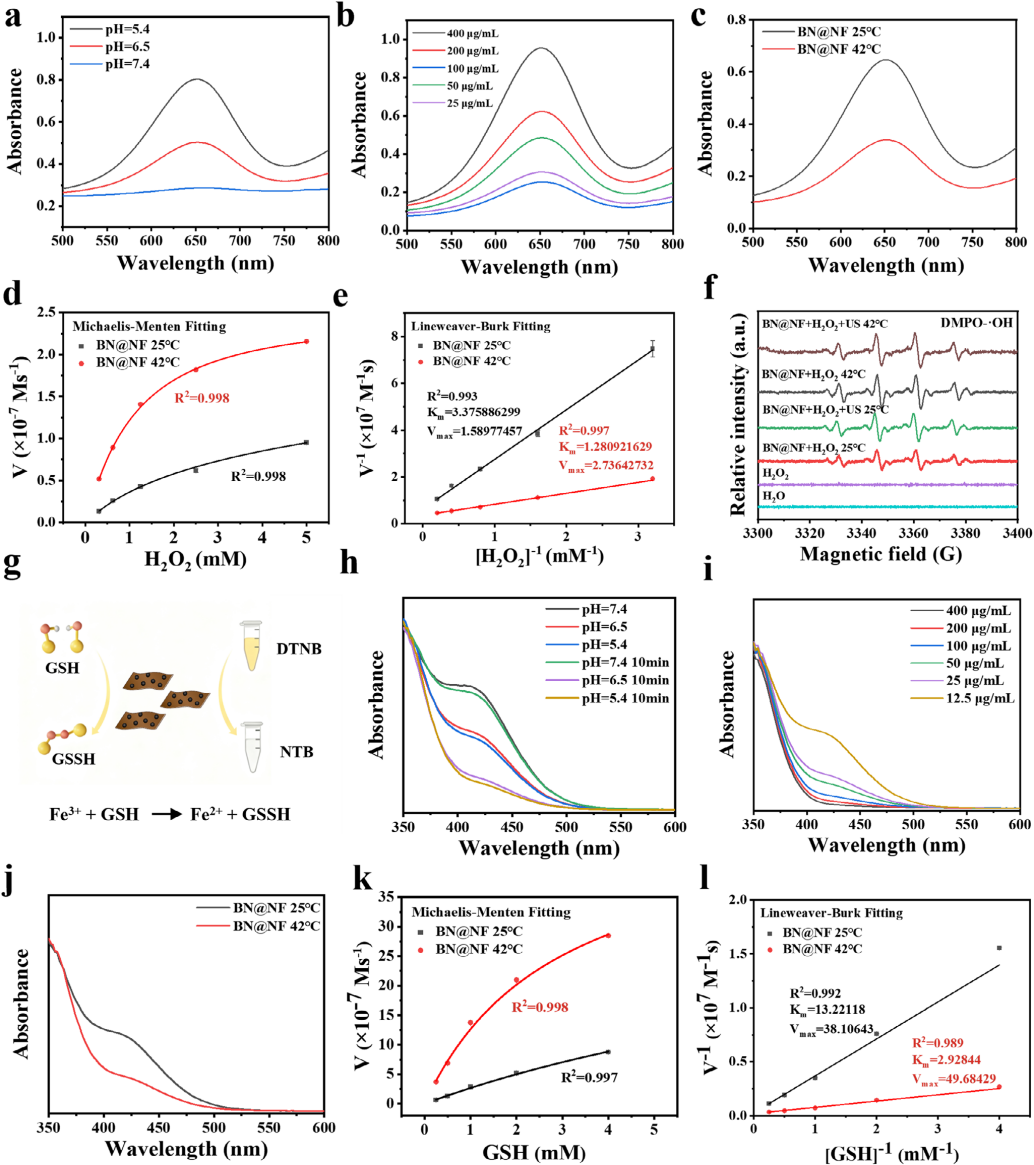
In vitro POD‐like and GSHOx‐like enzymatic activities of BN@NF. (a) TMB oxidation spectra demonstrating •OH generation catalyzed by BN@NF via H_2_O_2_ decomposition at varying pH levels. (b) TMB oxidation spectra showing •OH generation catalyzed by BN@NF at varying concentrations (pH = 5.4). (c) Comparative TMB oxidation spectra for •OH generation by BN@NF under different temperature (25°C and 42°C) at pH 5.4. (d) Michaelis–Menten kinetic analysis and (e) corresponding Lineweaver–Burk plots for BN@NF at 25°C and 42°C. (f) ESR spectroscopy characterization of •OH radical generation from H_2_O_2_ catalyzed by BN@NF. Measurements were conducted under varying temperatures (25°C and 42°C) with or without US irradiation (1.0 MHz, 1.0 W/cm^2^). (g) Schematic illustration of the GSH oxidation mechanism by BN@NF. (h) DTNB absorption spectra quantifying GSH depletion by BN@NF after 10 min incubation across pH values. (i) DTNB absorption spectra showing GSH depletion by BN@NF at different concentrations (pH = 5.4). (j) DTNB absorption spectra, (k) Michaelis–Menten kinetics, and (l) Lineweaver–Burk plots for GSH depletion by BN@NF, measured at pH 5.4 and temperatures of 25°C vs. 42°C. Data are presented as mean ± SD. n = 3 (d,e,k,l).

The multi‐enzyme‐mimetic behavior of BN@NF, including its peroxidase‐like (POD‐like) and glutathione oxidase‐like (GSHOx‐like) activities, was systematically evaluated. The POD‐like activity was first examined by monitoring the oxidation of TMB in the presence of H_2_O_2_. After BN@NF was added, a characteristic absorption band of oxidized TMB appeared at approximately 652 nm, indicating that H_2_O_2_ was decomposed and •OH were generated through a Fenton‐like process. The catalytic signal was strongly affected by both pH and BN@NF concentration (Figure 3a,b). The highest absorbance was recorded at pH 5.4, whereas much weaker signals were obtained at pH 6.5 and 7.4, indicating that the reaction was favored under mildly acidic conditions. In addition, the absorbance was progressively increased when the BN@NF concentration was raised from 25 to 400 µg/mL, confirming concentration‐dependent •OH production. When the temperature was increased from 25°C to 42°C, a markedly stronger TMB oxidation signal was obtained (Figure 3c), indicating that the catalytic process was promoted under the magnetothermal condition. The catalytic efficiency was then quantified by Michaelis–Menten analysis using H_2_O_2_ as the substrate. At 25°C, the apparent K_m_ and V_max_ values were 3.38 mM and 1.59 × 10^−7^ M·s^−1^, respectively. When the temperature was increased to 42°C, the K_m_ value was reduced to 1.28 mM and the V_max_ value was increased to 2.74 × 10^−7^ M·s^−1^ (Figure 3d,e). These changes indicated that stronger substrate affinity and faster catalytic turnover were achieved at the higher temperature. The generation of •OH was further verified by ESR spectroscopy using DMPO as the spin‐trapping agent. No obvious DMPO‐•OH signal was detected in the H_2_O or H_2_O_2_ control groups. After BN@NF was introduced, a characteristic quartet signal was observed, confirming that •OH was generated from H_2_O_2_ decomposition. The signal was further intensified under US irradiation and was strongest in the BN@NF+H_2_O_2_+US group at 42°C (Figure 3f). These results demonstrated that US and magnetothermal stimulation cooperatively enhanced the POD‐like activity of BN@NF.

Because intracellular GSH was able to quench ROS and reduce the efficacy of oxidative antibacterial therapy, the GSH‐depleting capability of BN@NF was further investigated by the DTNB assay. As illustrated in Figure 3g, GSH was oxidized to oxidized glutathione (GSSG) during Fe^3+^/Fe^2+^ redox cycling, which provided a mechanistic basis for the GSHOx‐like activity of BN@NF. The DTNB spectra showed clear pH‐ and concentration‐dependent GSH depletion (Figure 3h,i). After 10 min of incubation, the lowest residual GSH signal was detected at pH 5.4, whereas weaker depletion was observed at pH 6.5 and 7.4, indicating that GSH oxidation was also favored in an acidic environment. Similarly, when the BN@NF concentration was increased from 12.5 to 400 µg/mL, the DTNB absorbance was continuously decreased, indicating more efficient GSH consumption at higher concentrations. A further reduction in the residual GSH signal was observed at 42°C compared with 25°C (Figure 3j), showing that the GSHOx‐like activity was also promoted under the magnetothermal condition. This trend was further supported by kinetic analysis. At 25°C, the apparent K_m_ and V_max_ values for GSH were 13.22 mM and 38.11 × 10^−7^ M·s^−1^, respectively. At 42°C, the K_m_ value was reduced to 2.93 mM and the Vmax value was increased to 49.68 × 10^−7^ M·s^−1^ (Figure 3k,l), indicating improved substrate affinity and faster GSH oxidation at the higher temperature. Taken together, dual catalytic functions were demonstrated for BN@NF under mildly acidic and externally stimulated conditions. GSH was depleted through Fe^3+^‐associated redox conversion, whereas H_2_O_2_ was catalytically converted into highly reactive •OH through Fe^2+^‐mediated reactions. Through the simultaneous amplification of ROS production and exhaustion of antioxidant defenses, sustained oxidative stress was generated. This cooperative catalytic effect was therefore considered highly beneficial for bacterial eradication in infected lesions, where a mildly acidic microenvironment and external‐field stimulation could be used to maximize antibacterial efficacy.

### 3.4 In Vitro Antibacterial and Anti‐Biofilm Performance of BN@NF

The in vitro antibacterial activity of BN@NF was first optimized by using MDR‐*K.p* under combined AMF and US stimulation. As shown in Figure S8a, the number of bacterial colonies was progressively reduced as the BN@NF concentration was increased from 1 to 4 mg/mL, and this trend was observed at initial inocula of 10^3^, 10^2^, and 10 CFU/mL. For the 10^2^ CFU/mL group, the relative bacterial viability was reduced to approximately 60%, 22%, and 17% after treatment with 1, 2, and 4 mg/mL BN@NF, respectively (Figure S8b). Because comparable antibacterial efficacy was achieved at 2 and 4 mg/mL, 2 mg/mL was selected for the subsequent experiments to reduce the risk of nonspecific tissue injury caused by excessive temperature elevation.

**FIGURE 4:**
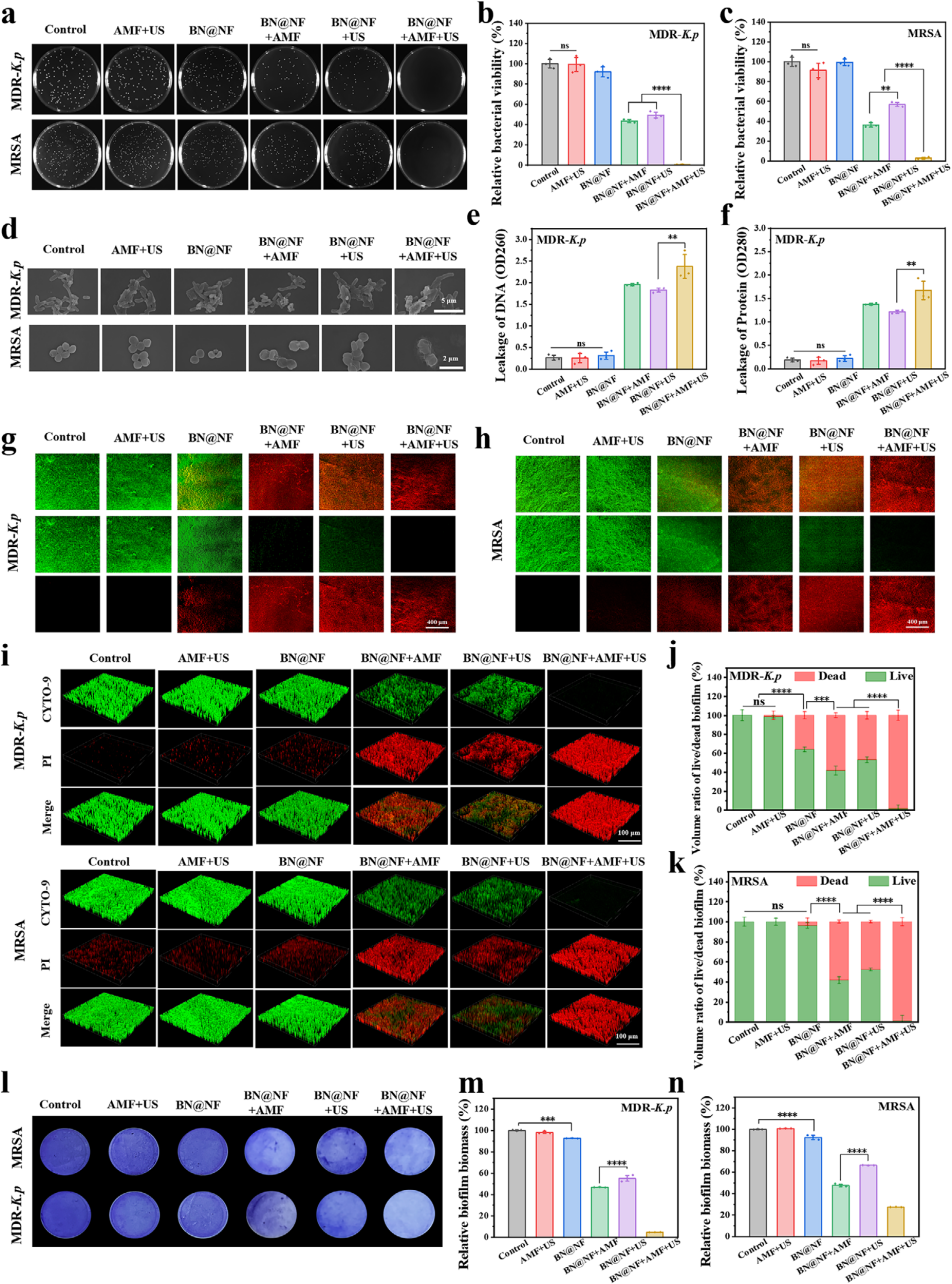
In vitro antibacterial and anti‐biofilm performance of BN@NF. (a) Plate spread images of MDR‐*K.p* and MRSA survival following treatment with BN@NF. (b,c) Quantitative analysis of bacterial survival rates. (d) SEM images of MRSA and MDR‐*K.p* cells. (e) Quantification of DNA leakage from MDR‐*K.p* bacteria. (f) Protein leakage analysis from MDR‐*K.p*. Fluorescence images depicting live/dead staining of MDR‐*K.p* (g) and MRSA (h) biofilms. (i) Three‐dimensional CLSM images and (j,k) corresponding fluorescence quantification of MRSA and MDR‐*K.p* biofilms. (l) CV staining of biofilms and (m, n) statistical analysis of residual biofilm biomass. The control group was the PBS treatment group. Results are shown as the average values ± S.D. (n = 3). **p < 0.01, ***p < 0.001, ****p < 0.0001, ns: not significant.

The antibacterial performance of BN@NF at 2 mg/mL was then evaluated against both MDR‐*K.p* and MRSA. As shown by the plate‐spread assays, little antibacterial effect was produced by AMF+US alone, and only limited colony reduction was observed in the BN@NF‐alone group (Figure 4a–c). After AMF activation, the relative viability of MDR‐*K.p* and MRSA was reduced to 43.50% and 36.45%, respectively. After US activation, the corresponding values were 49.33% and 57.01%. In contrast, under combined AMF and US stimulation, the antibacterial effect was markedly enhanced, and the killing rates reached 100% for MDR‐*K.p* and 97.07% for MRSA. These findings indicated that the bactericidal efficacy of BN@NF was strongly dependent on external activation. The pronounced enhancement observed under dual stimulation was attributed to the simultaneous amplification of catalytic ROS generation and the consumption of intracellular antioxidant components. The antibacterial mechanism was further examined by SEM and membrane leakage assays. In the control, AMF+US, and BN@NF groups, intact bacterial morphology was largely retained, and normal rod‐like MDR‐*K.p* and spherical MRSA cells were observed (Figure 4d). After activation by AMF or US, obvious structural damage was detected, including membrane shrinkage, collapse, and surface disruption. The most severe deformation was observed in the BN@NF+AMF+US group. This membrane injury was supported by the leakage results. For MDR‐*K.p*, DNA and protein leakage were both significantly increased after BN@NF activation, and the highest OD values at 260 and 280 nm were detected in the BN@NF+AMF+US group (Figure 4e,f). A similar trend was observed for MRSA, in which DNA and protein leakage were also most pronounced after dual stimulation (Figure S9). These results indicated that the bacterial membrane was severely compromised after BN@NF was activated, thereby promoting the leakage of intracellular biomacromolecules and accelerating bacterial death.

The anti‐biofilm performance of BN@NF was then evaluated because biofilm formation greatly limits bacterial eradication in pulmonary infections. Live/dead fluorescence staining showed that the control and AMF+US groups were dominated by green fluorescence in both MDR‐*K.p* and MRSA biofilms, indicating that most bacteria remained viable (Figure 4g,h). After treatment with BN@NF alone, partial red fluorescence was observed, suggesting only moderate biofilm killing. In contrast, markedly increased red fluorescence and reduced green fluorescence were detected in the BN@NF+AMF and BN@NF+US groups. The strongest effect was observed in the BN@NF+AMF+US group, in which the biofilms appeared predominantly red. Three‐dimensional confocal laser scanning microscopy (CLSM) further confirmed this trend (Figure 4i). Quantitative analysis showed that, for MDR‐*K.p* biofilms, the live fraction remained close to the control level in the AMF+US group, decreased to approximately 84% after BN@NF alone, fell to about 5% after BN@NF+AMF, and to about 28% after BN@NF+US, and was nearly eliminated after BN@NF+AMF+US treatment (Figure 4j and Figure S10a). For MRSA biofilms, the live fraction was reduced to approximately 76%, 34%, 40%, and 5% in the BN@NF, BN@NF+AMF, BN@NF+US, and BN@NF+AMF+US groups, respectively (Figure 4k and Figure S10b). These findings demonstrated that dual stimulation not only enhanced planktonic bacterial killing but also markedly improved the penetration and inactivation of established biofilms. The anti‐biofilm effect was further validated by CV staining and biomass quantification (Figure 4l‐n). Dense biofilm biomass was retained in the control and AMF+US groups, and only limited biomass reduction was observed after treatment with BN@NF alone. In contrast, clear biofilm disruption was observed after BN@NF was activated by AMF or US, and the lowest residual biomass was detected in the BN@NF+AMF+US group. Quantitative analysis showed that the residual biofilm biomass was reduced to 4.66% for MDR‐*K.p* and 27.44% for MRSA after dual stimulation. The higher residual biomass of MRSA suggested that its biofilm structure was more resistant to disruption than that of MDR‐*K.p*. Taken together, these results demonstrated that BN@NF possessed intrinsic antibacterial activity, but its full antibacterial and anti‐biofilm potential was achieved only after external activation. Under combined AMF and US stimulation, membrane destruction, intracellular leakage, bacterial death, and biofilm removal were all markedly intensified. This controllable US‐magnetic activation pattern therefore provided strong support for the use of BN@NF as a catalytic antibacterial platform for bacterial pneumonia.

### 3.5 In Vitro Assessment of Anti‐Inflammatory Effects and Mitophagy Enhancement of BN@NF

**FIGURE 5:**
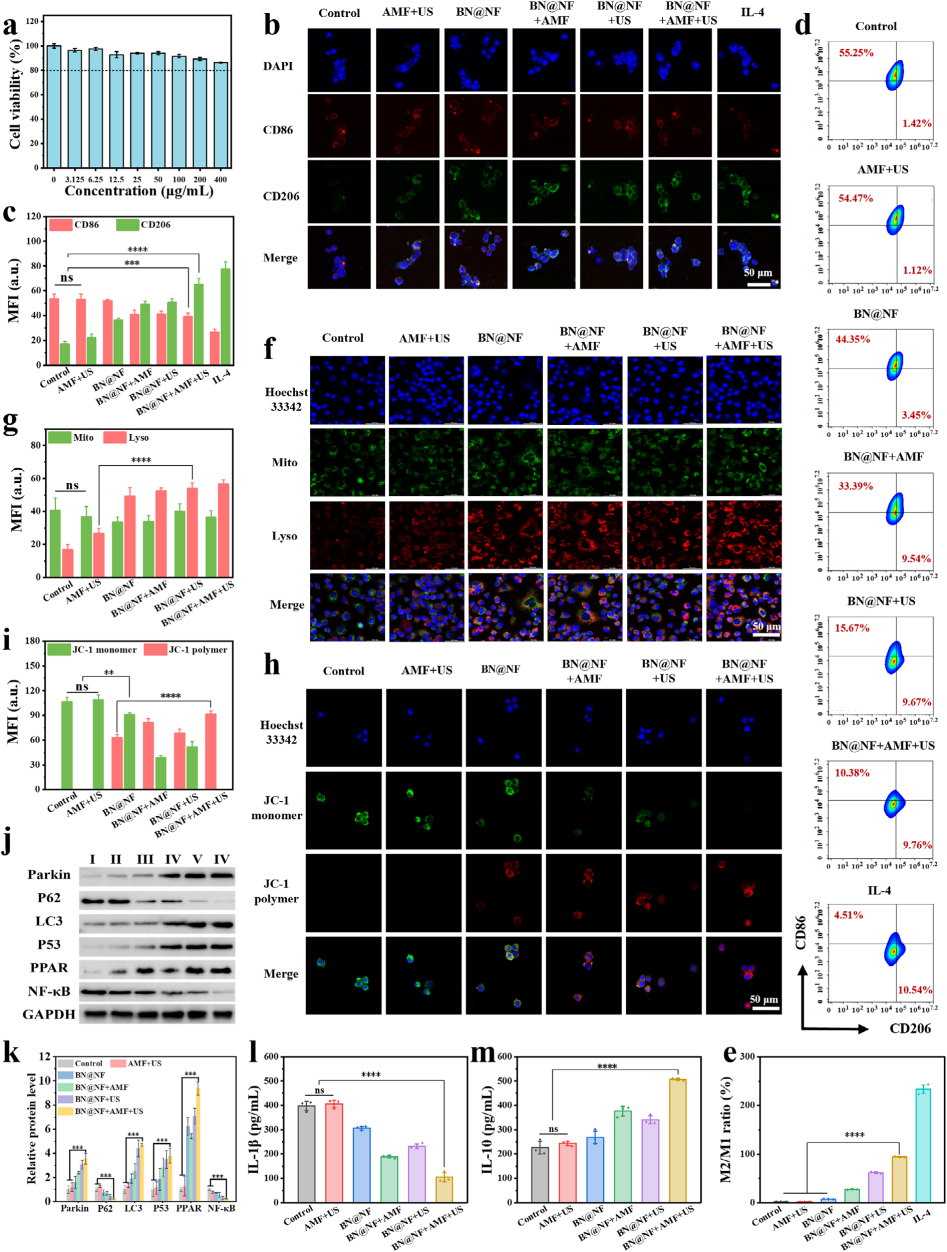
In vitro evaluation of BN@NF‐induced mitophagy enhancement and anti‐inflammatory effects in RAW264.7 macrophages. (a) Cell viability of RAW264.7 cells treated with various concentrations of BN@NF. (b) Representative immunofluorescence images of CD86 and CD206 expression in RAW264.7 cells following different treatments. (c) Quantification of the MFI of CD86 (red) and CD206 (green) in RAW264.7 cells. (d) Flow cytometry analysis of macrophage polarization showing CD86 and CD206 expression in different treatment groups. (e) M2/M1 macrophage polarization ratio analysis by flow cytometry. (f) Confocal fluorescence images of mitochondrial (MitoTracker^TM^ Green) and lysosomal (LysoTracker^TM^ Red) staining in treated RAW264.7 cells. (g) Quantification of MFI of mitochondria and lysosomes in different treatment groups. (h) Confocal fluorescence images illustrating mitochondrial membrane potential (JC‐1 staining) in treated RAW264.7 cells. (i) Quantification of MFI of JC‐1 monomer and polymer in different treatment groups. (j) Western blot analysis of mitophagy‐related proteins (Parkin, P62, LC3, P53, PPAR, and NF‐κB) in RAW264.7 cells after treatment with different stimuli (I, Control; II, AMF+US; III, BN@NF; IV, BN@NF+AMF; V, BN@NF+US; VI, BN@NF+AMF+US). (k) Quantification of relative protein levels of mitophagy‐related markers. Levels of (l) IL‐1β and (m) IL‐10 in the supernatant of treated RAW264.7 cells. PBS treatment was used as the control group. Data are presented as a mean ± S.D. (n = 3). **p < 0.01, ***p < 0.001, ****p < 0.0001, ns: not significant.

To determine whether BN@NF could regulate macrophage inflammation while maintaining acceptable cytocompatibility, RAW264.7 cells were first incubated with increasing concentrations of BN@NF. As shown in Figure 5a, cell viability gradually decreased as the BN@NF concentration increased, but it remained above 85% even at 400 µg/mL. These results indicated that BN@NF exhibited acceptable cytocompatibility within the tested concentration range. Therefore, the subsequent cell‐based experiments were considered unlikely to be dominated by nonspecific cytotoxicity. To assess the anti‐inflammatory activity of BN@NF, an inflammatory macrophage model was established by LPS stimulation. Because bacterial pneumonia is commonly associated with excessive M1 macrophage accumulation, the effects of BN@NF on macrophage polarization were examined by immunofluorescence staining and flow cytometry. As shown in Figure 5b–e, strong CD86 fluorescence and weak CD206 fluorescence were observed in the control and AMF+US groups, indicating that external stimulation alone did not reverse the pro‐inflammatory phenotype. After treatment with BN@NF alone, CD86 expression was reduced and CD206 expression was increased, suggesting that partial M1‐to‐M2 repolarization was induced. This trend became more pronounced after AMF or US activation and was most evident in the BN@NF+AMF+US group, in which CD86 staining was markedly weakened and CD206 staining was markedly enhanced, approaching the IL‐4‐positive control. Flow cytometry further supported these observations. In the control group, 55.25% of cells were CD86‐positive, whereas only 1.42% were CD206‐positive. Similar values were obtained in the AMF+US group (54.47% and 1.12%, respectively). After treatment with BN@NF alone, these values shifted to 44.35% and 3.45%. After BN@NF+AMF treatment, they further changed to 33.39% and 9.54%, and after BN@NF+US treatment, they changed to 15.67% and 9.67%. The strongest polarization shift was observed in the BN@NF+AMF+US group, in which the proportion of CD86‐positive cells decreased to 10.38% and the proportion of CD206‐positive cells increased to 9.76%. Consistently, the M2/M1 ratio was markedly increased in the BN@NF+AMF+US group and approached the level of the IL‐4 group. These findings indicated that BN@NF, particularly under dual stimulation, efficiently redirected macrophages from a pro‐inflammatory state toward an anti‐inflammatory phenotype.

To investigate whether the observed immunoregulatory effect was linked to mitophagy activation, mitochondria‐lysosome colocalization was assessed by MitoTracker and LysoTracker staining. As demonstrated in Figure 5f,g, mitochondrial fluorescence changed only slightly among the groups, whereas lysosomal fluorescence was markedly increased after BN@NF treatment. More importantly, the merged fluorescence signal became progressively stronger in the BN@NF+AMF, BN@NF+US, and BN@NF+AMF+US groups, indicating enhanced mitochondria‐lysosome colocalization. Quantitative analysis showed that lysosomal mean fluorescence intensity (MFI) increased from approximately 16 a.u. in the control group to about 50, 52, 55, and 57 a.u. in the BN@NF, BN@NF+AMF, BN@NF+US, and BN@NF+AMF+US groups, respectively. In contrast, the mitochondrial signal remained within a relatively narrow range. These results suggested that lysosomal recruitment to damaged mitochondria was promoted by BN@NF treatment and was further strengthened by AMF and US stimulation, which was consistent with enhanced mitophagy.

To determine whether BN@NF‐mediated mitophagy was associated with mitochondrial functional recovery, mitochondrial membrane potential (ΔΨm) was further assessed by the JC‐1 assay after carbonyl cyanide m‐chlorophenyl hydrazone (CCCP) treatment. In the control and AMF+US groups, JC‐1 was mainly present in its monomeric form, as indicated by dominant green fluorescence, confirming marked ΔΨm depolarization (Figure 5h). After treatment with BN@NF alone, a partial shift from green to red fluorescence was observed. This shift became more evident after AMF or US activation and was strongest in the BN@NF+AMF+US group, indicating the recovery of ΔΨm. Quantitative analysis showed that the JC‐1 monomer signal was reduced, whereas the JC‐1 polymer signal was increased, in the BN@NF‐containing groups, with the largest change detected after dual stimulation (Figure 5i). These findings suggested that damaged mitochondria were cleared more efficiently after BN@NF treatment, thereby contributing to the restoration of mitochondrial homeostasis.

The molecular basis of this effect was next examined by western blotting. As shown in Figure 5j,k, Parkin and LC3 expression was upregulated in the BN@NF‐related groups, whereas P62 expression showed a declining trend, particularly after AMF and/or US activation. These changes were consistent with enhanced autophagic and mitophagic flux. Parkin is a central regulator of PINK1/Parkin‐dependent mitophagy [68, 69], whereas LC3 lipidation and P62 degradation are widely recognized indicators of autophagosome formation and cargo turnover [70]. In addition, P53 expression showed an increasing trend together with Parkin, suggesting that P53 might participate in mitophagy activation in this model [71]. PPAR expression was also increased, especially in the BN@NF+AMF+US group, whereas NF‐κB expression was reduced. Because PPAR has been reported to interfere with inflammatory signaling and to antagonize NF‐κB activation under certain conditions [72], these results suggested that a P53/PPAR/NF‐κB‐associated axis might have contributed to the anti‐inflammatory effect of BN@NF.

This interpretation was further supported by cytokine analysis (Figure 5l,m). The level of interleukin‐1β (IL‐1β) remained high in the control and AMF+US groups at approximately 400 pg/mL, decreased to approximately 310 pg/mL after BN@NF treatment alone, and was further reduced to approximately 190, 235, and 105 pg/mL after BN@NF+AMF, BN@NF+US, and BN@NF+AMF+US treatment, respectively. In contrast, the level of interleukin‐10 (IL‐10) increased from approximately 220 pg/mL in the control group to approximately 280, 380, 345, and 505 pg/mL in the BN@NF, BN@NF+AMF, BN@NF+US, and BN@NF+AMF+US groups, respectively. These results indicated that BN@NF suppressed the release of pro‐inflammatory cytokines while promoting the production of anti‐inflammatory cytokines, and that the strongest immunomodulatory effect was achieved under combined AMF and US stimulation. Taken together, these results demonstrated that BN@NF exerted dual regulatory effects on macrophages after external activation. On the one hand, mitophagy was enhanced, as evidenced by increased mitochondria‐lysosome colocalization, recovery of ΔΨm, upregulation of Parkin and LC3, and reduction of P62. On the other hand, inflammatory signaling was attenuated, as reflected by the suppression of NF‐κB and IL‐1β, the increase in PPAR and IL‐10, and the marked shift from M1 to M2 polarization. The strongest effects were consistently observed in the BN@NF+AMF+US group. These findings suggested that BN@NF alleviated macrophage inflammatory activation by restoring mitochondrial homeostasis and promoting pro‐resolving phenotypic reprogramming, which was highly favorable for the treatment of bacterial pneumonia.

### 3.6 In Vivo Biodistribution and Therapeutic Efficacy Assessment of BN@NF in Pneumonia

**FIGURE 6:**
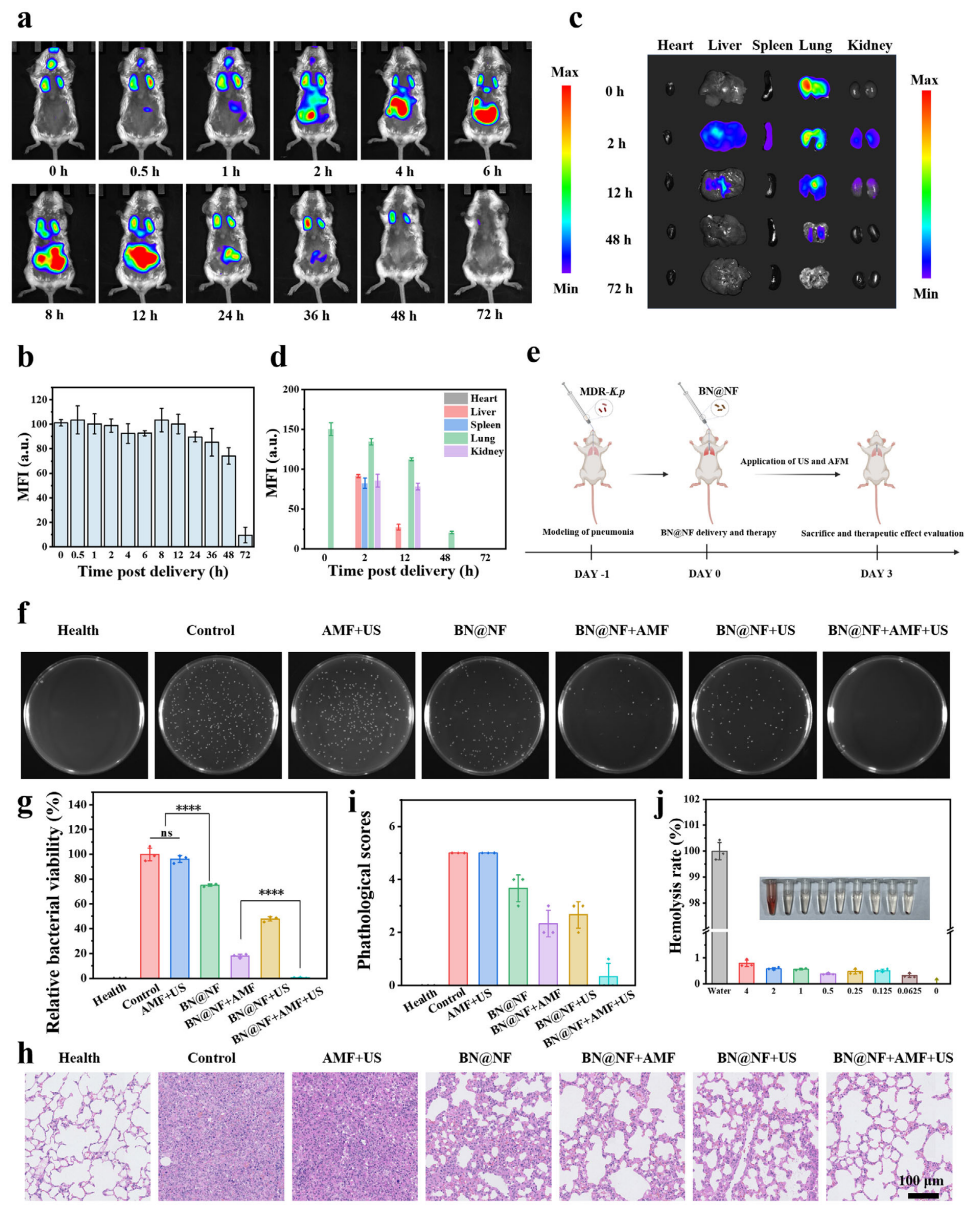
In vivo biodistribution and therapeutic evaluation of nebulized BN@NF in a murine bacterial pneumonia model. (a) Representative whole‐body fluorescence images acquired at 0, 0.5, 1, 2, 4, 6, 8, 12, 24, 36, 48, and 72 h post‐delivery. (b) Quantification of fluorescence signal intensity (MFI; a.u.) at the indicated time points (n = 6). (c) Ex vivo fluorescence images of major organs (heart, liver, spleen, lung, and kidney) collected at 0, 2, 12, 48, and 72 h post‐delivery. (d) Quantification of organ fluorescence signals (MFI; a.u.) at the indicated time points (n = 6). (e) Schematic of the in vivo study timeline, including pneumonia establishment using MDR‐*K.p* (d ‐1), BN@NF administration and treatment (d 0), and sample collection/endpoint assessment (d 3), with US and AMF applied as indicated. (f) Representative bacterial colony plates prepared from lung homogenates for the indicated groups (Health, Control, AMF+US, BN@NF, BN@NF+AMF, BN@NF+US, and BN@NF+AMF+US). (g) Relative bacterial viability (%) quantified from colony counts for each group (n = 3). (h) Representative hematoxylin and eosin (H&E) staining of lung sections from each group (scale bar, 100 µm). (i) Pathological scores derived from lung histology (n = 3). (j) Hemolysis rate (%) measured after incubation with BN@NF at the indicated concentrations (n = 3), with representative photographs of the hemolysis assay tubes shown as an inset. ****p < 0.0001, ns: not significant.

To investigate the in vivo biodistribution of BN@NF after inhalation delivery, BN@NF was labeled with ICG and administered to mice by nebulization. Whole‐body fluorescence imaging showed that the signal was predominantly localized to the thoracic region immediately after administration, indicating rapid deposition in the respiratory tract (Figure 6a,b). The fluorescence intensity remained relatively high during the first 12 h, with MFI values close to 100 a.u., and then gradually declined to approximately 89 a.u. at 24 h and 74 a.u. at 48 h. By 72 h, the signal had decreased to near‐background levels. These results indicated that BN@NF was effectively delivered to the lungs and retained for a sufficient period to support subsequent therapy. This distribution pattern was further confirmed by ex vivo imaging of major organs collected at 0, 2, 12, 48, and 72 h after delivery (Figure 6c,d). The strongest fluorescence signal was detected in the lungs immediately after administration, with an MFI of approximately 150 a.u. at 0 h, which remained high at about 135 a.u. at 2 h and 112 a.u. at 12 h. In contrast, the heart showed minimal fluorescence throughout the observation period. Transient signals were also detected in the liver, spleen, and kidneys, particularly at 2 h, suggesting partial systemic redistribution and subsequent clearance through hepatic and renal pathways. By 48 h, only weak fluorescence remained in the lungs, and almost no signal was detected in any organ at 72 h. Together, these findings demonstrated that inhaled BN@NF achieved lung‐dominant accumulation followed by gradual systemic clearance.

The therapeutic efficacy of BN@NF was then evaluated in a murine pneumonia model established with MDR‐*K.p*, according to the schedule shown in Figure 6e. On d 3, the lungs were harvested, homogenized, and plated for bacterial enumeration. As shown in Figure 6f,g, abundant bacterial colonies were recovered from the control group, and a similarly high bacterial burden was observed in the AMF+US group, indicating that physical stimulation alone did not exert an obvious antibacterial effect in vivo. After treatment with BN@NF alone, the relative bacterial viability was reduced to approximately 76%, indicating moderate intrinsic antibacterial activity. After AMF activation, the relative bacterial viability decreased further to about 18%, whereas a value of approximately 48% was observed after US activation. The strongest therapeutic effect was obtained in the BN@NF+AMF+US group, in which almost no colonies were detected and the relative bacterial viability approached 0%. These findings demonstrated that dual‐field activation markedly enhanced the in vivo antibacterial efficacy of BN@NF. The therapeutic benefit of BN@NF was further supported by lung histopathology. In the healthy group, intact alveolar structures and thin alveolar septa were observed (Figure 6 h). In contrast, severe pathological damage was detected in the control and AMF+US groups, including extensive inflammatory cell infiltration, alveolar wall thickening, and marked disruption of normal lung architecture. After treatment with BN@NF alone, the pathological injury was partially alleviated, but obvious inflammatory lesions were still present. More substantial recovery was observed in the BN@NF+AMF and BN@NF+US groups, as reflected by reduced inflammatory infiltration and improved alveolar morphology. The most pronounced histological improvement was observed in the BN@NF+AMF+US group, in which the lung structure was largely restored and closely resembled that of the healthy group. Consistently, the pathological score remained at approximately 5.0 in the control and AMF+US groups, decreased to about 3.6 after BN@NF alone, fell further to about 2.3 and 2.7 in the BN@NF+AMF and BN@NF+US groups, respectively, and reached approximately 0.4 in the BN@NF+AMF+US group (Figure 6i and Table S2). These results indicated that bacterial clearance by activated BN@NF was accompanied by marked attenuation of pneumonia‐associated lung injury.

The hemocompatibility of BN@NF was also assessed to support its in vivo application. As shown in Figure 6j, water induced complete hemolysis, whereas BN@NF caused only minimal hemolysis over the tested concentration range of 0–4 mg/mL. The hemolysis rate remained below 1% at all concentrations, which was well below the commonly accepted 5% safety threshold. These data indicated that BN@NF exhibited acceptable blood compatibility under the experimental conditions. To further evaluate systemic safety, histological, biochemical, and hematological analyses were performed after treatment. H&E staining of the heart, liver, spleen, and kidneys showed no obvious treatment‐related lesions, inflammatory infiltration, or structural destruction in any BN@NF‐treated group (Figure S11a). Serum biochemical parameters, including albumin (ALB), alanine aminotransferase (ALT), aspartate aminotransferase (AST), urea (UREA), creatinine (CREA), and uric acid (UA), did not show progressive deterioration after BN@NF treatment, with or without external stimulation (Figure S11b–g). Likewise, peripheral blood indices, including lymphocytes, white blood cells, platelets, red blood cells, hemoglobin, and mean corpuscular volume, did not show a pattern consistent with treatment‐induced hematological toxicity (Figure S11h–m). Collectively, these findings demonstrated that BN@NF achieved effective lung‐targeted antibacterial therapy with acceptable in vivo biosafety.

### 3.7 Anti‐inflammatory Mechanism of BN@NF in a Pneumonia Mouse Model

**FIGURE 7:**
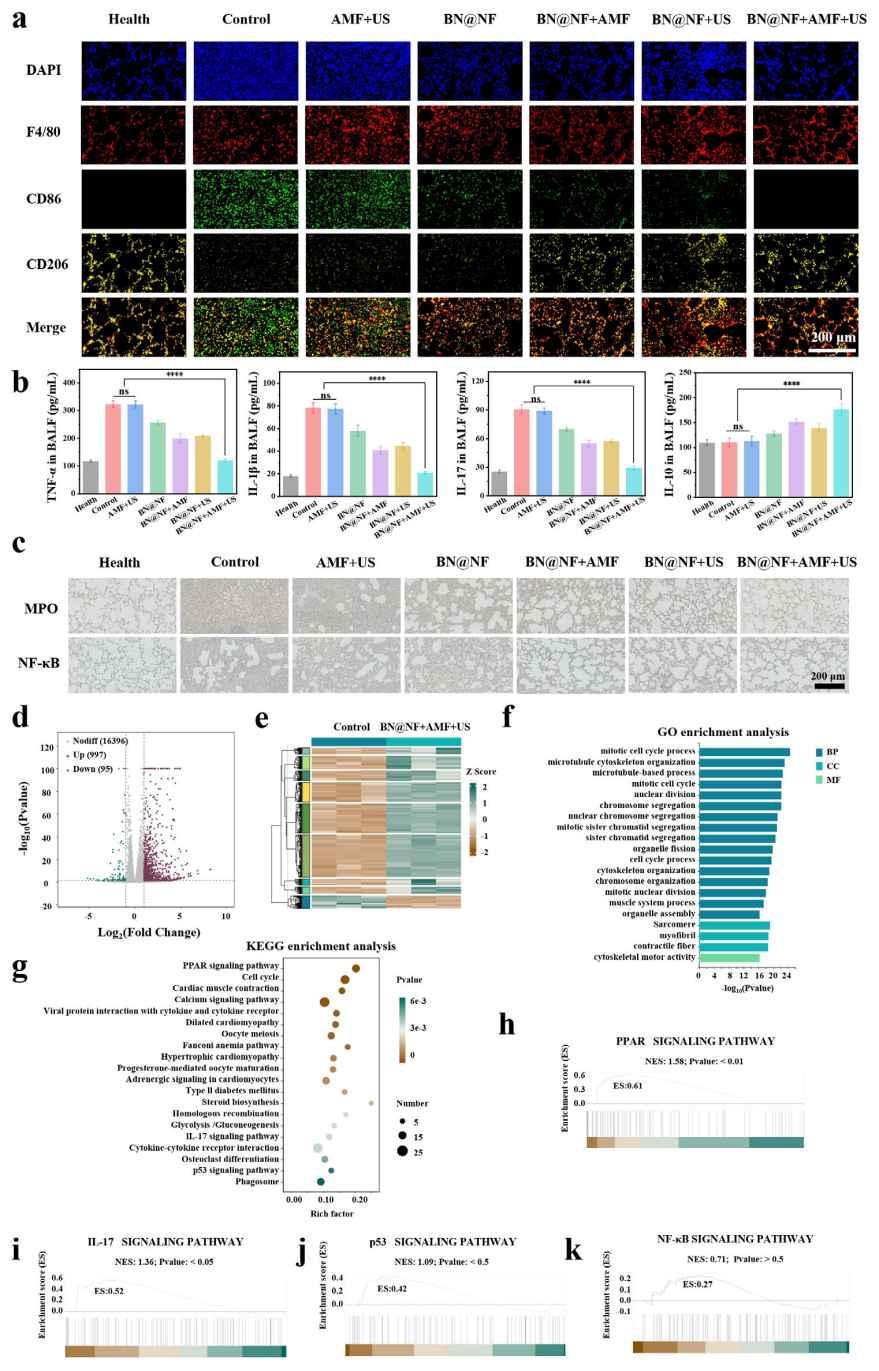
In vivo inflammation modulation and gene expression analysis of BN@NF in a murine bacterial pneumonia model. (a) Representative immunofluorescence images showing the expression of F4/80, CD86, and CD206 in lung tissues from different treatment groups, with DAPI staining for nuclei. Scale bar, 200 µm. (b) Levels of key cytokines (TNF‐α, IL‐1β, IL‐17, IL‐10) in BALF from different treatment groups post‐therapy. (c) Representative immunohistochemistry (IHC) images of MPO and NF‐κB expression in lung tissues. Scale bar, 200 µm. (d) Volcano plot showing the differential expression of genes between the untreated Control and BN@NF+AMF+US groups. (e) Heatmap of DEGs in the no‐treatment Control and BN@NF+AMF+US groups. (f) GO enrichment analysis for the DEGs, categorizing them into biological processes (BP), cellular components (CC), and molecular functions (MF). (g) KEGG enrichment analysis for DEGs, identifying enriched pathways such as PPAR signaling and cell cycle regulation. GSEA plots depicting enrichment of the (h) PPAR signaling pathway, (i) IL‐17 signaling pathway, (j) p53 signaling pathway, and (k) NF‐κB signaling pathway within the KEGG database. PBS treatment was used as the control group. Values represent mean ± S.D. (n = 6). ****p < 0.0001. ns, no significant difference.

Following the demonstrated in vivo antibacterial efficacy and therapeutic benefit of BN@NF in MDR‐*K.p*‐induced pneumonia, its anti‐inflammatory activity and underlying mechanism were further evaluated. Immunofluorescence staining was first performed on lung sections to assess macrophage polarization. As shown in Figure 7a, F4/80^+^ macrophages were abundantly detected in the inflamed lungs of the Control and AMF+US groups, where strong CD86 staining and weak CD206 staining were observed, indicating the persistence of an M1‐dominant inflammatory phenotype. After treatment with BN@NF alone, CD86 fluorescence was reduced and CD206 fluorescence was moderately increased, suggesting partial macrophage repolarization. This trend became more evident after AMF or US activation. The most pronounced phenotypic shift was observed in the BN@NF+AMF+US group, in which CD86^+^ macrophages were markedly decreased and CD206^+^ macrophages were markedly increased, approaching the distribution observed in healthy lung tissue. These findings indicated that BN@NF alleviated pulmonary inflammation in part by promoting macrophage polarization toward an M2‐like phenotype in vivo.

This phenotypic transition was accompanied by a marked change in cytokine secretion in both BALF and serum. As shown in Figure 7b, TNF‐α, IL‐1β, and IL‐17 levels in BALF were sharply elevated in the Control group and remained at similar levels in the AMF+US group, indicating that physical stimulation alone did not relieve the inflammatory response. Specifically, TNF‐α, IL‐1β, and IL‐17 remained at approximately 320, 80, and 90 pg/mL, respectively, in these two groups. After treatment with BN@NF alone, these cytokines were reduced to approximately 255, 60, and 70 pg/mL, respectively. Greater reductions were observed after external activation, with values of approximately 195, 43, and 55 pg/mL in the BN@NF+AMF group and approximately 205, 46, and 58 pg/mL in the BN@NF+US group. The lowest levels were detected in the BN@NF+AMF+US group, in which TNF‐α, IL‐1β, and IL‐17 decreased to approximately 110, 20, and 30 pg/mL, respectively, approaching the healthy baseline. In contrast, the anti‐inflammatory cytokine IL‐10 showed the opposite pattern. Its level remained near 110 pg/mL in the Health, Control, and AMF+US groups, increased to approximately 126 pg/mL after BN@NF treatment alone, and further rose to approximately 152, 138, and 178 pg/mL in the BN@NF+AMF, BN@NF+US, and BN@NF+AMF+US groups, respectively. A consistent trend was also observed in serum (Figure S12). In the BN@NF+AMF+US group, serum TNF‐α, IL‐1β, and IL‐17 decreased to approximately 112, 19, and 26 pg/mL, respectively, whereas IL‐10 increased to approximately 160 pg/mL. These results demonstrated that BN@NF not only attenuated local pulmonary inflammation, but also reshaped the systemic inflammatory profile toward a resolution‐associated state. The suppression of inflammatory injury was further confirmed by immunohistochemical analysis of myeloperoxidase (MPO) and NF‐κB in lung tissues. MPO, which is mainly localized in neutrophil granules, is widely used as an indicator of neutrophil infiltration and activation status in inflamed tissues [73]. As shown in Figure 7c, strong MPO and NF‐κB staining was observed in the Control and AMF+US groups, indicating severe neutrophilic inflammation and persistent activation of pro‐inflammatory signaling. After treatment with BN@NF, both staining signals were progressively weakened, and the lowest expression levels were detected in the BN@NF+AMF+US group. These findings indicated that activated BN@NF reduced inflammatory cell recruitment and attenuated NF‐κB‐associated inflammatory signaling in vivo.

To further clarify the molecular basis of this anti‐inflammatory effect, transcriptomic profiling was performed on lung tissues from the untreated Control group and the BN@NF+AMF+US group. As shown in the volcano plot, 1,092 differentially expressed genes (DEGs) were identified, including 997 upregulated and 95 downregulated genes in the BN@NF+AMF+US group, whereas 16,396 genes were not significantly changed (Figure 7d). The heatmap further showed clear clustering separation between the two groups, indicating reproducible treatment‐induced transcriptional remodeling (Figure 7e). Gene Ontology (GO) enrichment analysis showed that the DEGs were mainly associated with mitotic cell cycle process, organelle fission, organelle assembly, microtubule‐based process, chromosome segregation, and related structural programs (Figure 7f). These results suggested that, in addition to inflammation control, active tissue remodeling and cellular turnover were induced during the recovery phase of pneumonia. Further pathway analysis provided additional mechanistic insight. Kyoto Encyclopedia of Genes and Genomes (KEGG) analysis further revealed enrichment of pathways related to peroxisome proliferator‐activated receptor (PPAR) signaling, cell cycle regulation, cytokine‐cytokine receptor interaction, IL‐17 signaling, p53 signaling, and phagosome function (Figure 7g). Gene set enrichment analysis (GSEA) showed significant enrichment of the PPAR signaling pathway (normalized enrichment score, NES = 1.58; p < 0.01) and the IL‐17 signaling pathway (NES = 1.36; p < 0.05) (Figure 7h,i). In contrast, only weak enrichment trends were observed for the p53 pathway (NES = 1.09; p < 0.5) and the NF‐κB pathway (NES = 0.71; p > 0.5) (Figure 7j,k). Therefore, strong transcriptomic support was provided for activation of a PPAR‐associated regulatory program, whereas transcriptome‐wide support for p53‐ and NF‐κB‐centered changes remained limited. When these results were interpreted together with the cytokine, immunofluorescence, and immunohistochemistry data, the anti‐inflammatory mechanism of BN@NF was most consistently associated with macrophage repolarization, suppression of pro‐inflammatory cytokine output, and activation of a PPAR‐related repair program.

## 4 Conclusion

In this study, the inhalable BN@NF nanozyme provided an integrated therapeutic strategy for bacterial pneumonia by coupling efficient pathogen eradication with active inflammation control. Through the Z‐scheme heterostructure and the coordinated action of US and AMF stimulation, BN@NF strengthened ROS generation, promoted H_2_O_2_ utilization and GSH depletion, disrupted bacterial membranes and biofilms, and achieved strong antibacterial activity against drug‐resistant pathogens both in vitro and in vivo. At the same time, BN@NF alleviated pneumonia‐associated inflammatory injury by suppressing NF‐κB‐related signaling, enhancing mitophagy, restoring mitochondrial homeostasis, reducing pro‐inflammatory cytokines, and shifting macrophages from an M1‐dominant state toward an M2 reparative phenotype. These findings support the central hypothesis that effective treatment of bacterial pneumonia requires not only bacterial clearance but also precise regulation of the inflammatory microenvironment, and they establish a multi‐responsive catalytic platform that links magneto‐sono‐enhanced nanozyme activity with immunometabolic reprogramming. Compared with conventional antibiotic‐centered approaches and previously reported ROS‐based antibacterial systems that mainly emphasize direct microbial killing, this work achieved simultaneous control of infection, biofilm burden, mitochondrial stress, and immune imbalance while maintaining favorable lung retention and biosafety after nebulized delivery. Future studies should focus on long‐term safety, dosing optimization, large‐animal validation, and broader testing in complex infection settings, including mixed‐species biofilms and chronic pneumonia models, to further advance the translational potential of this externally activatable nanozyme system.

## Author Contributions

**Xiaofeng Luo**: methodology, investigation, data curation, writing – original draft. **Yundi Wu**: methodology, investigation, formal analysis, data curation, writing – original draft. **Shuai Zhang**: methodology, validation. **Huanran Qu**: formal analysis, data curation. **Shiyang Shao**: methodology, validation. **Jianqiang Chen**: formal analysis, data curation. **Xilong Wu**: conceptualization, funding acquisition, resource, supervision, writing – reviewing and editing.

## Conflicts of Interest

The authors declare that they have no known competing financial interests or personal relationships that could have appeared to influence the work reported in this paper.

## Supporting information




**Supporting File**: advs77785‐sup‐0001‐SuppMat.pdf.

## Data Availability

The data that support the findings of this study are available from the corresponding author upon reasonable request.
