# Supplementary material for: Magneto‐Sono‐Enhanced Nanozyme Catalysis Drives ROS‐Mediated Pathogen Killing and Mitophagy‐Linked Inflammation Control in Bacterial Pneumonia
Source: Adv Sci (Weinh). 2026 Sep 16:e77785. Online ahead of print. doi: 10.1002/advs.77785 (PMC13583241; doi:10.1002/advs.77785)
Supplement: Supplementary file 1 [file ADVS-9999-e77785-s001.pdf]

Magneto-Sono-Enhanced Nanozyme Catalysis Drives ROS-Mediated Pathogen Killing and Mitophagy-Linked Inflammation Control in Bacterial Pneumonia

Xiaofeng Luo [1,2†], Yundi Wu [1,2,3,4†], Shuai Zhang [1,2], Huanran Qu [1,2,5], Shiyang Shao [5], Jianqiang Chen [4], Xilong Wu [1,2*]

[1] State Key Laboratory of Digital Medical Engineering, School of Biomedical Engineering, The Affiliated Sanya Central Hospital of Hainan University, School of Pharmaceutical Sciences, Hainan University, Sanya, China

[2] Collaborative Innovation Center of One Health, Key Laboratory of Biomedical Engineering of Hainan Province, Hainan University, Haikou, China

[3] Key Laboratory of Tropical Biological Resources of Ministry of Education and Hainan Engineering Research Center for Drug Screening and Evaluation, Hainan University, Haikou, China

[4] Engineering Research Center for Hainan Biological Sample Resources of Major Diseases, Key Laboratory of Emergency and Trauma of Ministry of Education, The First Affiliated Hospital, Hainan Medical University, Haikou, China

[5] School of Materials Science and Engineering, Hainan University, Haikou, China

[†]These authors contributed equally to this work.

[*]**Corresponding author**

E-mail: xilongwu@foxmail.com (X. Wu)

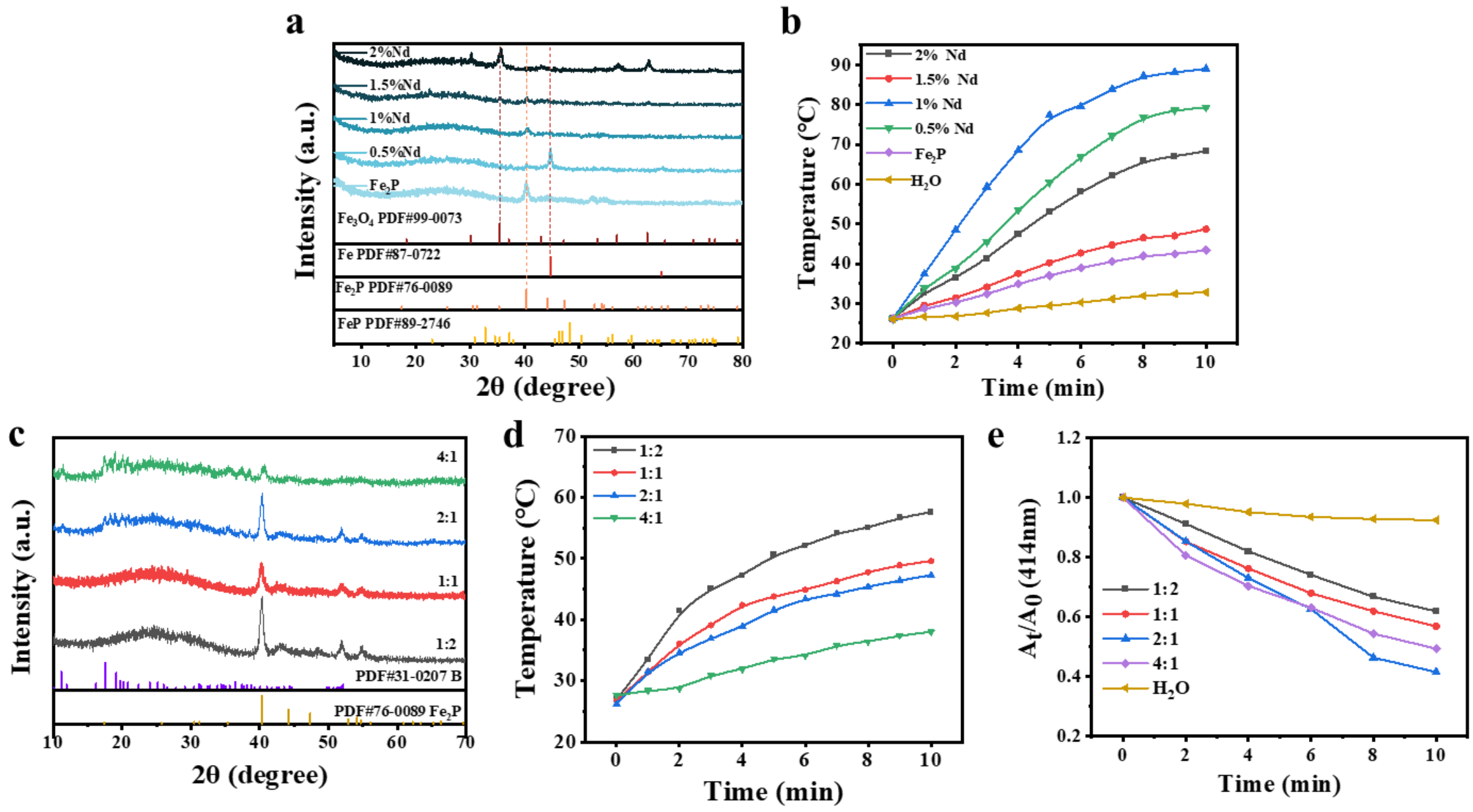

Figure S1. Characterization and performance of Nd-doped Fe_2P nanocomposites for heat generation and catalytic activity. (a) X-ray diffraction (XRD) patterns of Nd-doped Fe_2P nanocomposites with varying Nd concentrations (0.5%, 1%, 1.5%, 2%) compared with Fe_2P. (b) Temperature increase of Nd-doped Fe_2P nanocomposites in response to an alternating magnetic field (AMF) at varying Nd concentrations (0.5%, 1%, 1.5%, 2%) over a 10-min period. (c) XRD patterns of BN@NF nanocomposites with different BN:NF ratios (1:2, 1:1, 2:1, 4:1). (d) Temperature increase of BN@NF nanocomposites under AMF exposure at different BN:NF ratios (1:2, 1:1, 2:1, 4:1) over 10 min. (e) DPBF degradation profiles at varying BN@NF ratios (1:2, 1:1, 2:1, 4:1) over a 10-min period under AMF.

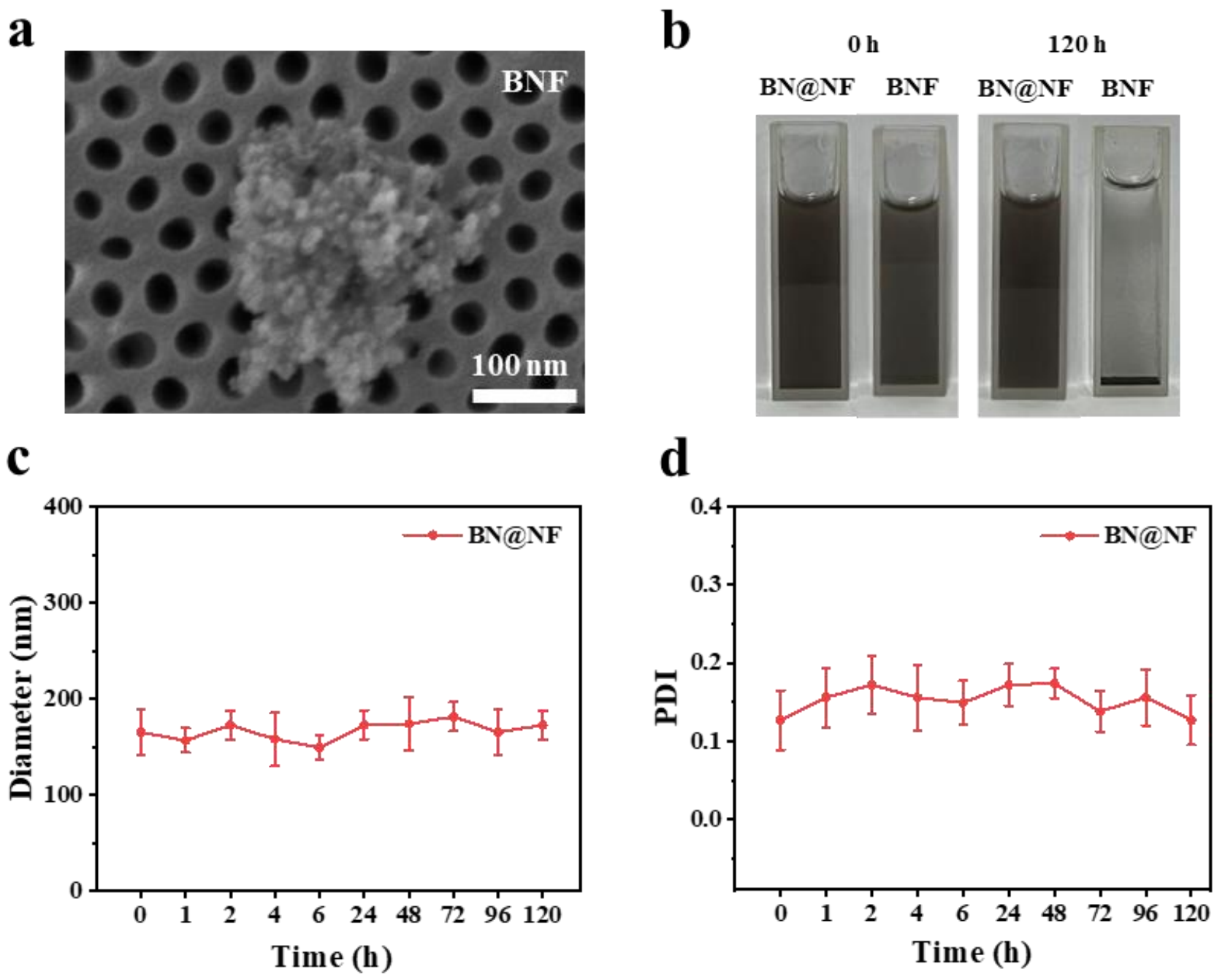

Figure S2. (a) SEM image of BNF. (b) Photographs showing the dispersion of BNF and BN@NF at 0 h and 120 h. (c) Size variation of BNF over a period of 120 h. (d) Variation in PDI (polydispersity index) of BNF over 120 h. Data are presented as a mean ± S.D. (n = 3).

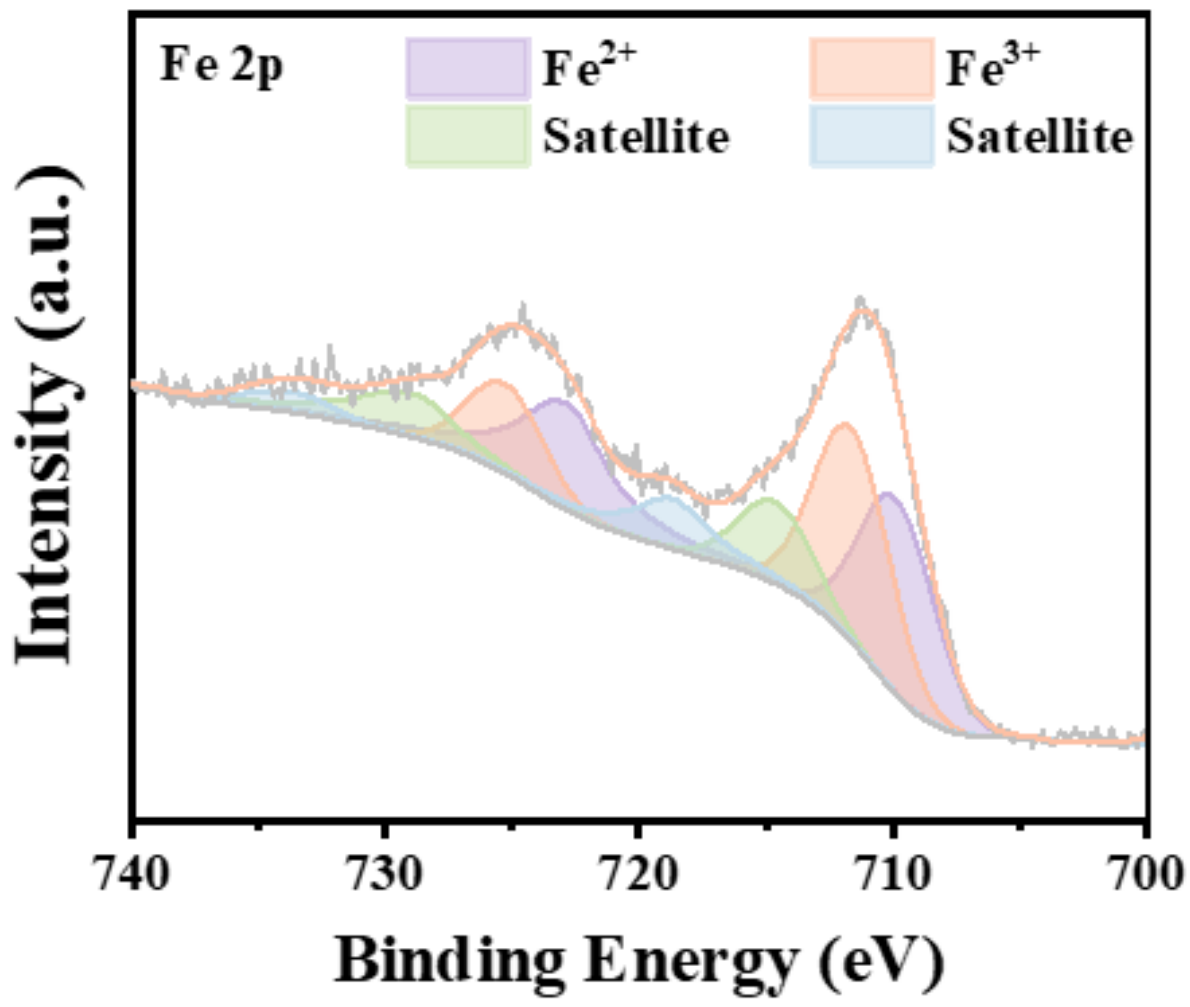

Figure S3. High-resolution XPS spectrum of Fe 2p in BN@NF.

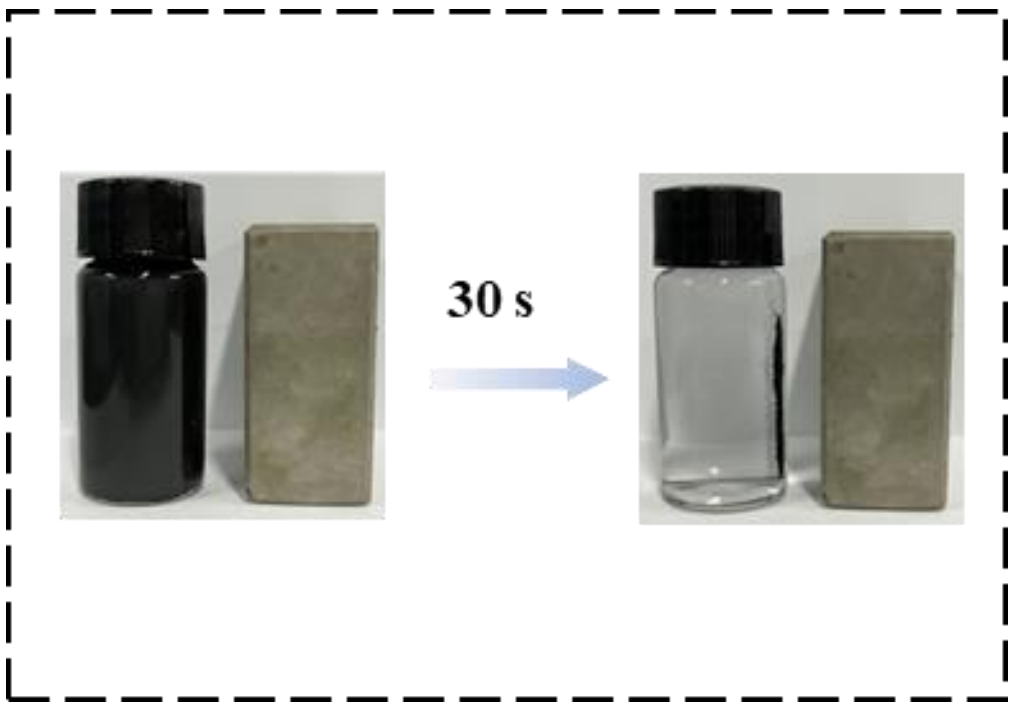

Figure S4. Photograph demonstrating the magnetic response of an aqueous solution of BN@NF under an applied magnetic field.

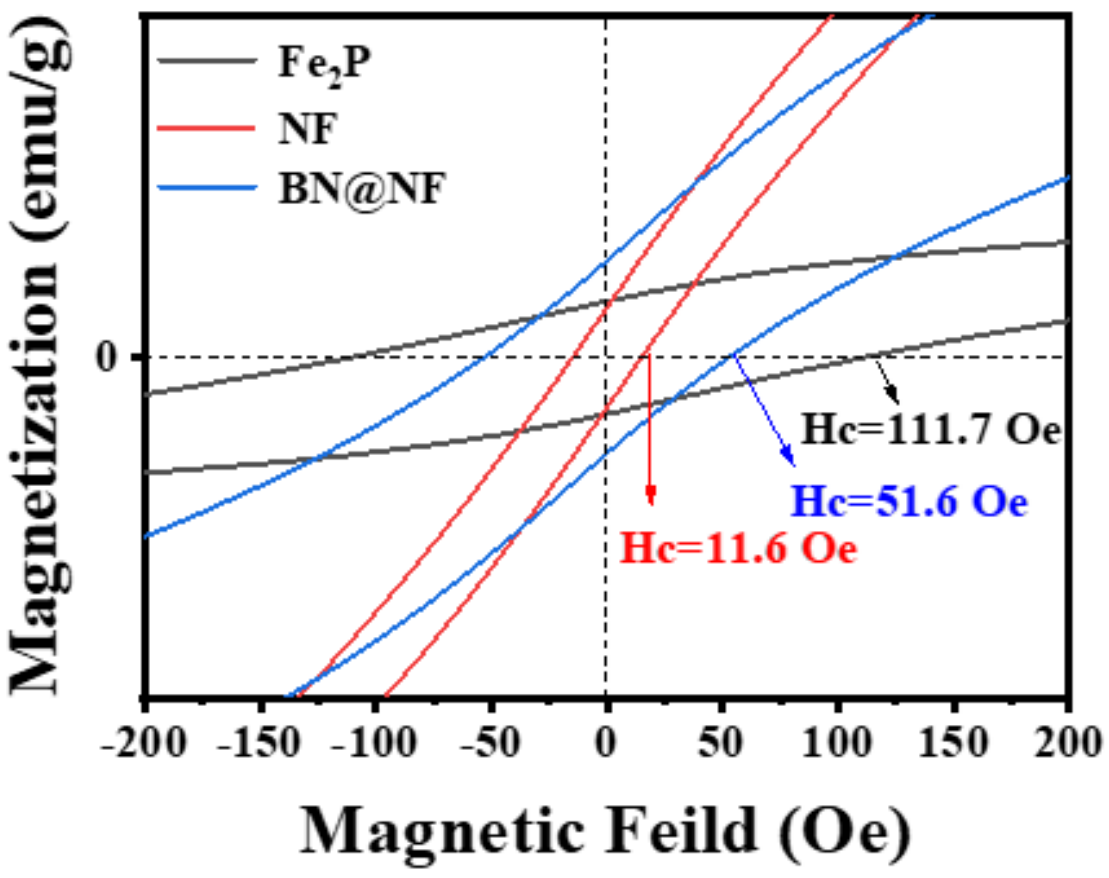

Figure S5. Magnified view of hysteresis loops for Fe_2P, NF, and BN@NF under applied magnetic field conditions.

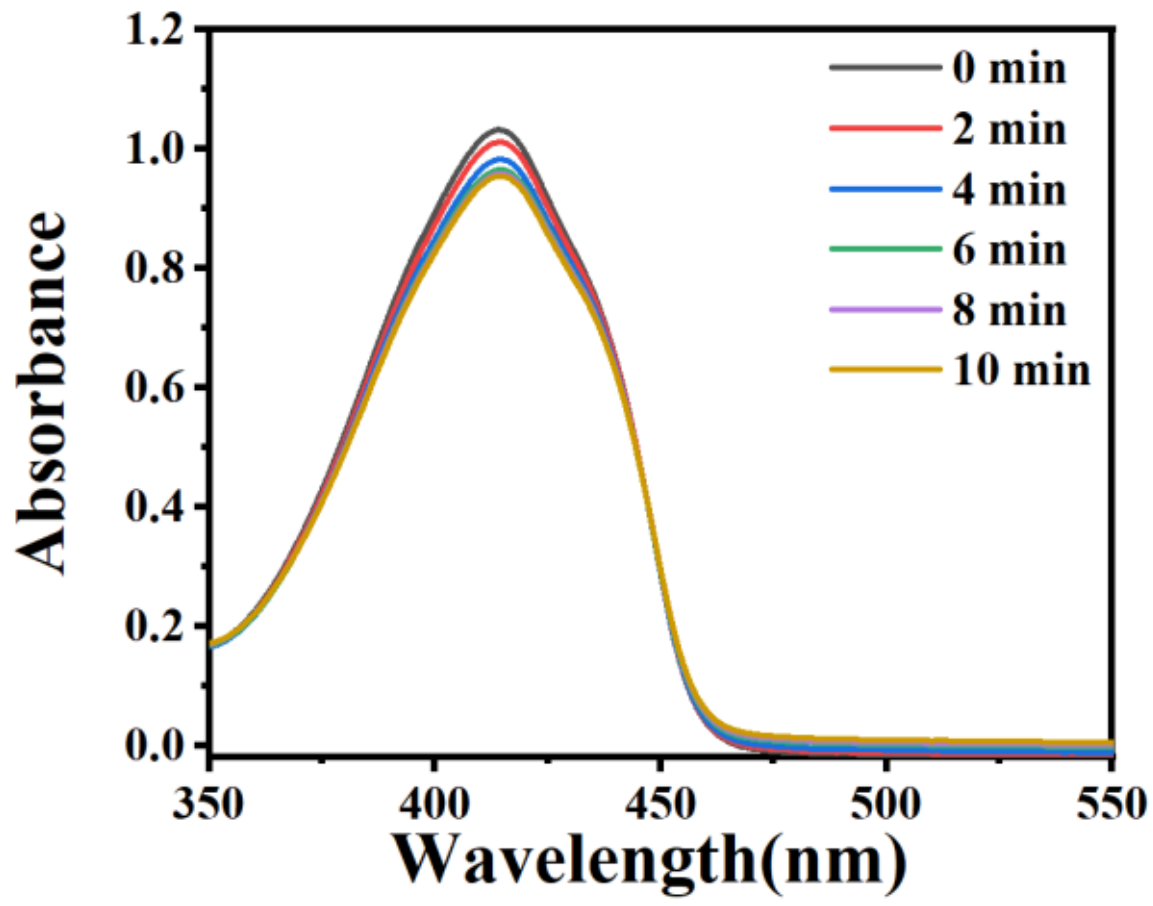

Figure S6. Time-dependent absorption spectra showing the degradation of the $^1O_2$ probe DPBF in aqueous solution under US irradiation (1.0 MHz, 1 W·cm^{-2}).

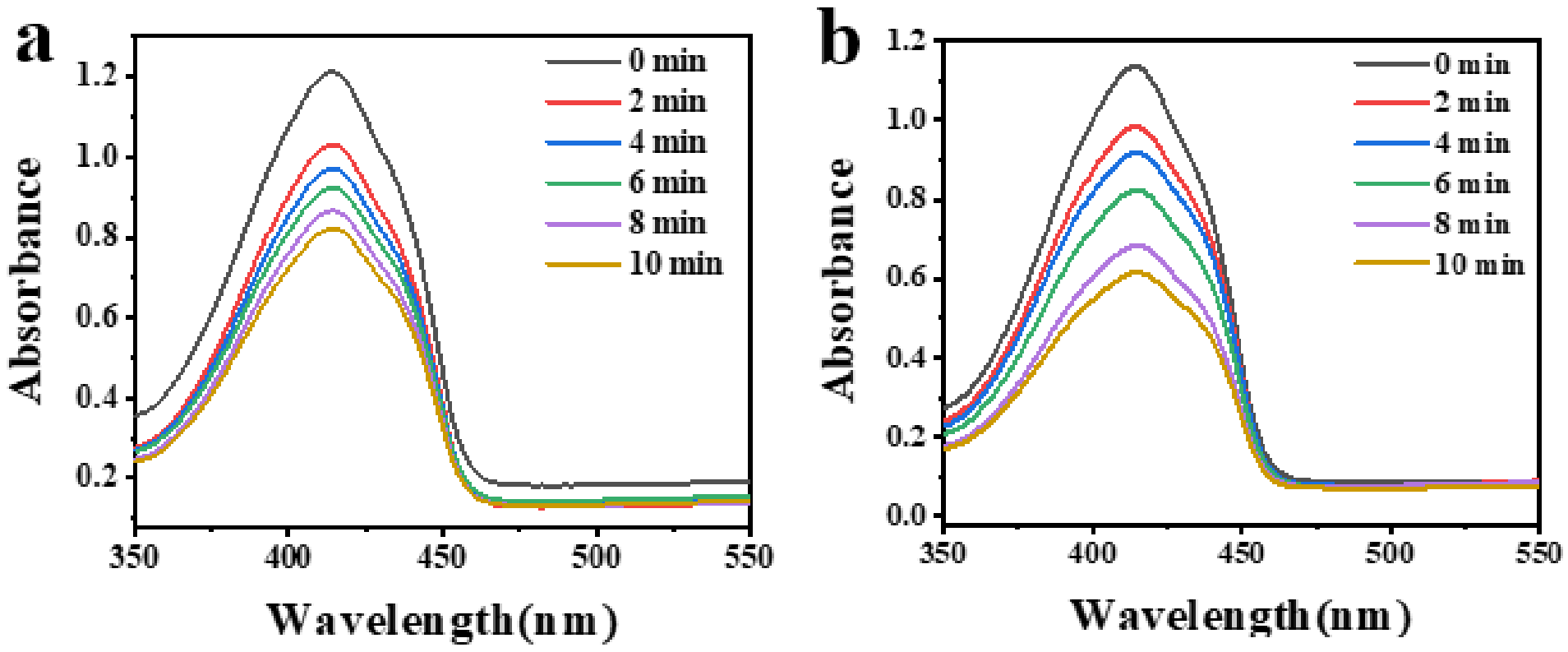

Figure S7. Time-dependent UV-Vis spectra of DPBF degradation by $^1O_2$ generated under US treatment (1.0 MHz, 1.0 W/cm^2) from (a) NF and (b) BN.

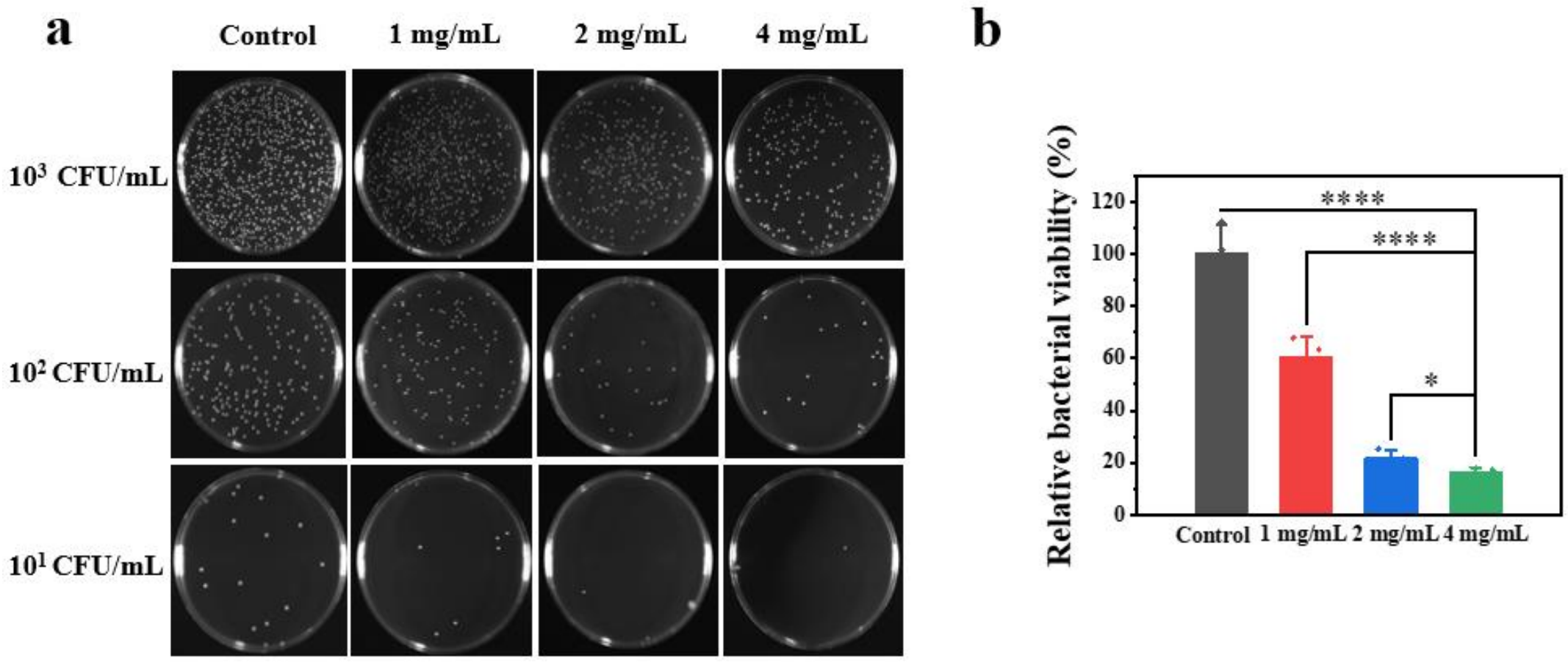

Figure S8. Concentration-dependent antibacterial activity of BN@NF under AMF and US against MDR-*K.p*. (a) Representative colony-forming unit (CFU) plate images of MDR-*K.p* after treatment with BN@NF at 1, 2, or 4 mg/mL (Control: PBS), using initial bacterial inocula of 10^3, 10^2, and 10^1 CFU/mL as indicated, followed by plating for CFU enumeration. (b) Relative bacterial viability (%) calculated from CFU counts for the 10^2 CFU/mL inoculum group. Values represent mean ± S.D. (n = 3). *$P < 0.05$, ****$P < 0.0001$.

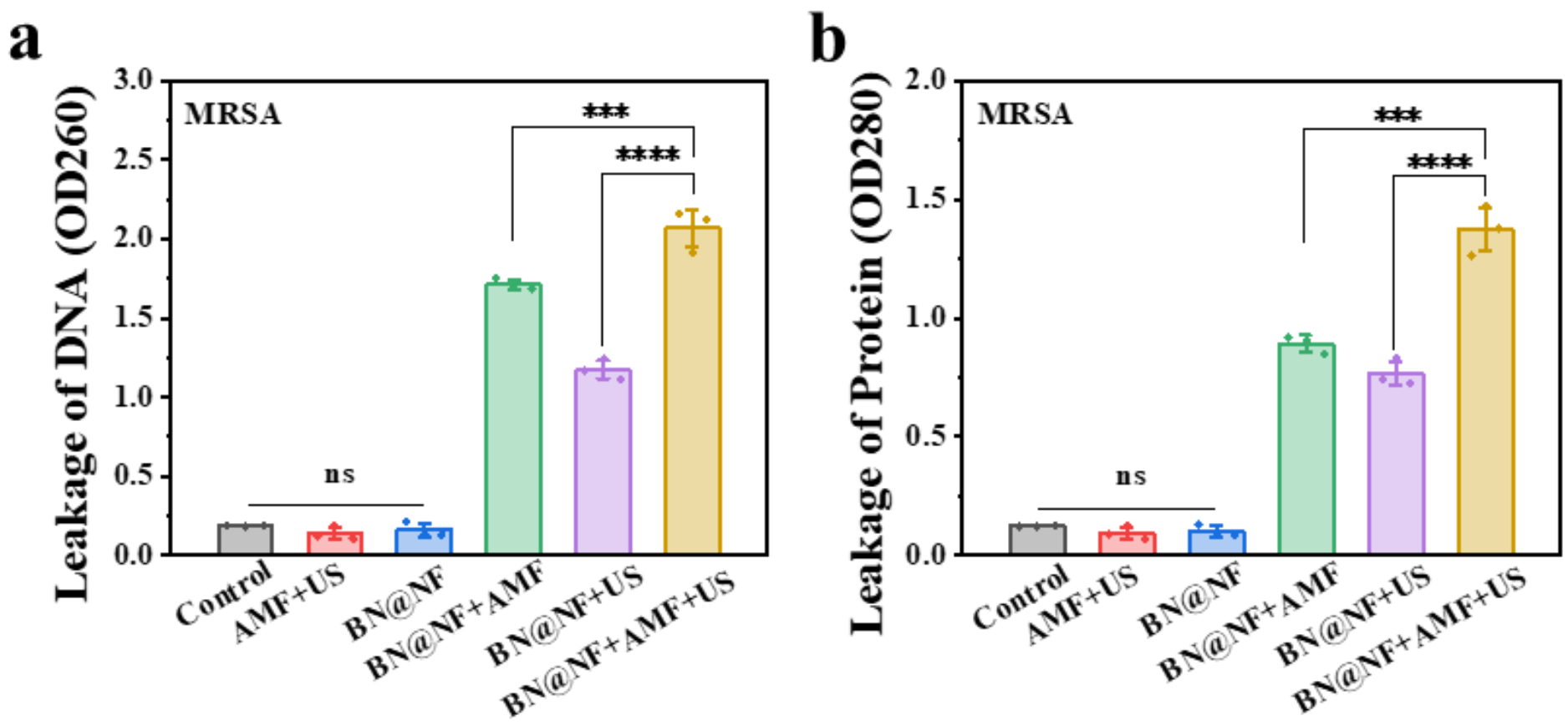

Figure S9. Quantification of (a) DNA and (b) protein leakage from MRSA bacteria following different treatments. Results are shown as the average values ± S.D. (n = 3). Control: PBS. ****P* < 0.001, *****P* < 0.0001, ns: not significant.

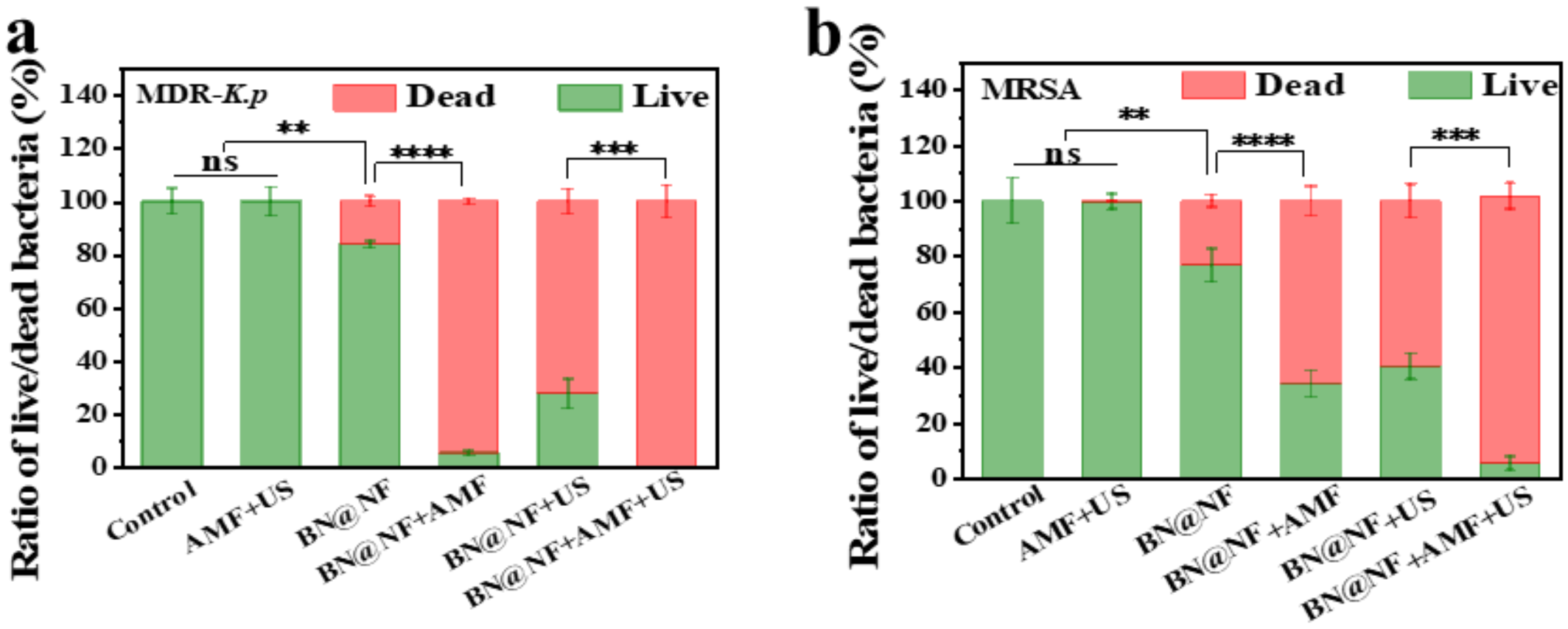

Figure S10. Quantitative analysis of live/dead staining for (a) MDR-*K.p* and (b) MRSA post-treatment. Values represent mean ± S.D. (n = 3). Control: PBS. $^{**}P < 0.01$, $^{***}P < 0.001$, $^{****}P < 0.0001$, ns: not significant.

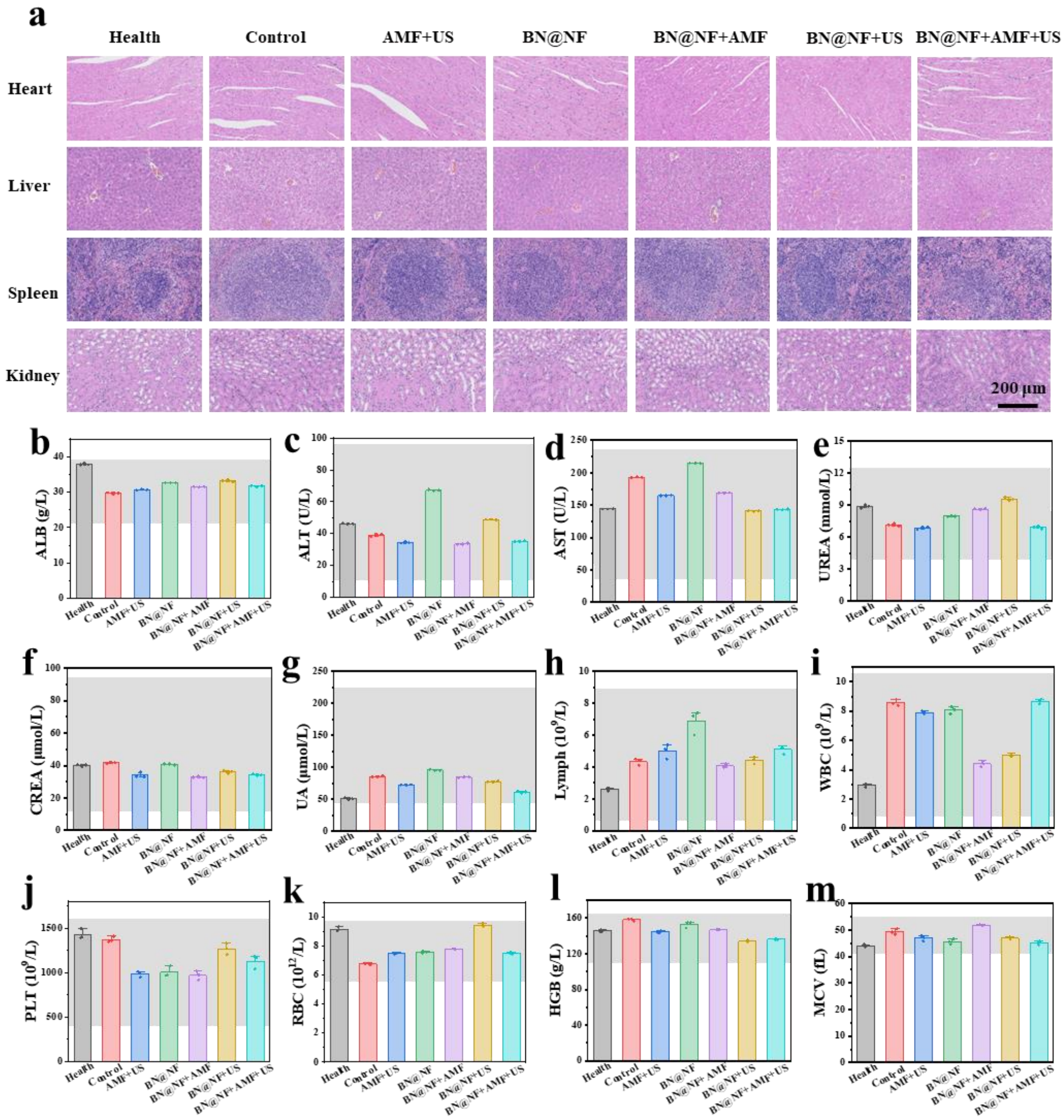

Figure S11. In vivo toxicity evaluation of BN@NF in a murine pneumonia model. (a) Representative H&E-stained sections of major organs (heart, liver, spleen, and kidneys) harvested from mice post-treatment. (b-g) Biochemical analysis of liver and kidney function markers in serum samples from different experimental groups post-treatment, including ALB, ALT, AST, UREA, CREA, and UA. (h-m) Hematological analysis of peripheral blood parameters across treatment groups post-treatment, including Lymph, WBC, PLT, RBC, HGB, and MCV. The control group was PBS treatment group. Data are presented as a mean ± S.D. (n = 3).

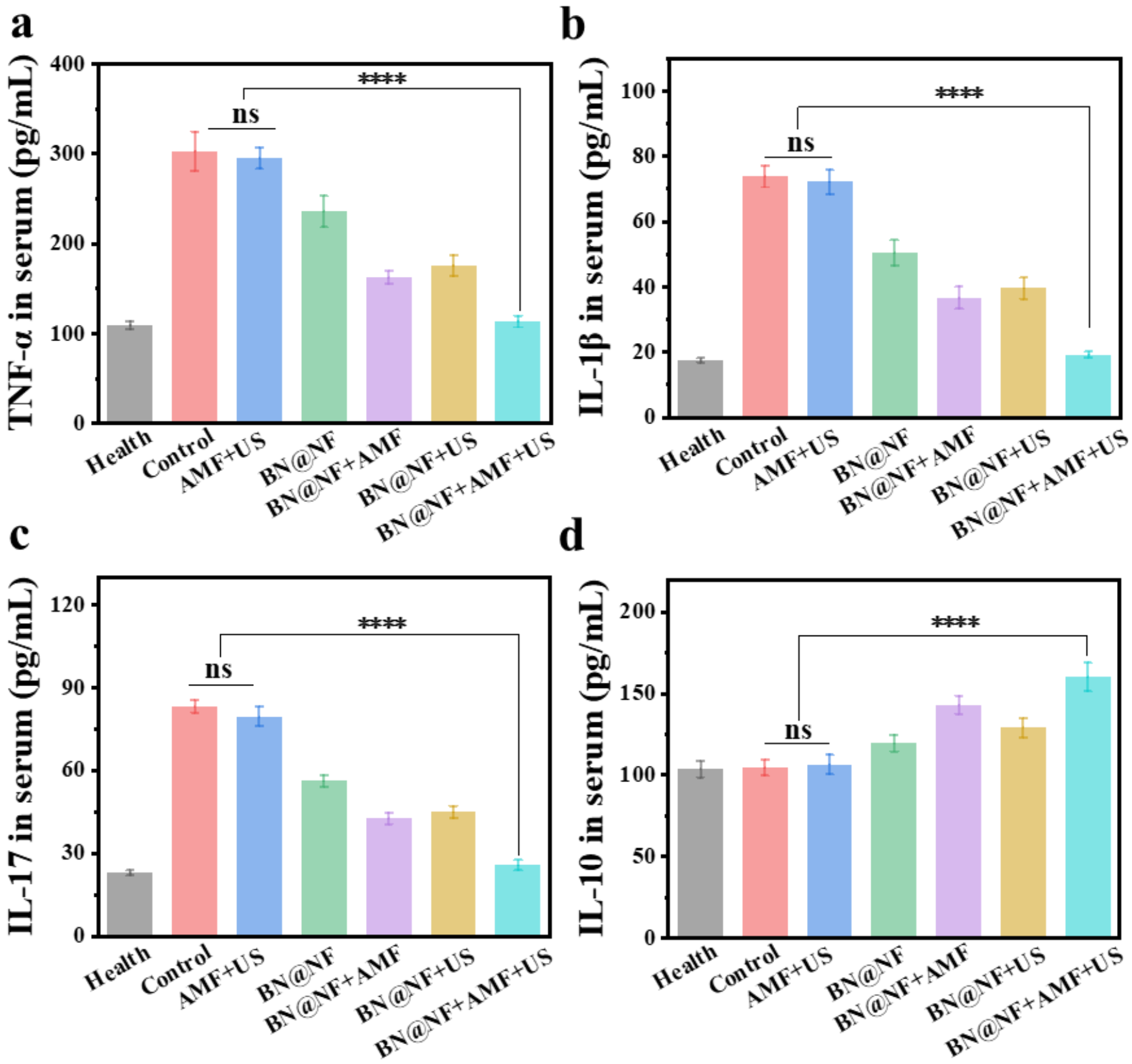

Figure S12. Serum levels of (a) TNF-α, (b) IL-1β, (c) IL-17, and (d) IL-10 cytokines in each group after treatment (n = 6). Control: PBS. ****$P < 0.0001$, ns: not significant.

Table S1. Specific Absorption Rate (SAR) values of Fe_2P, $Nd_{0.02}Fe_{1.98}P$, and BN@NF under AMF conditions.

Component	SAR（W/g）
Fe_2P	36.5
$Nd_{0.02}Fe_{1.98}P$	157.5
BN@NF	109.9

Table S2. Key for histopathology scoring of inflammation reaction in lung tissues post-treatment.

Score value	Description of degree of inflammation
0	No reaction in alveolar walls.
1	Diffuse reaction in alveolar walls, primarily neutrophilic, no thickening of alveolar walls.
2	Diffuse presence of inflammatory cells neutrophilic and mononuclear in alveolar walls with slight thickening.
3	Distinct（2-3 times） thickening of the alveolar walls due to presence of inflammatory cells.
4	Alveolar wall thickening with up to 25% of lung consolidated.
5	Alveolar wall thickening with more than 50% of lung consolidated.
